# *Limousia* bacteria encode mucinolysome for mucin utilization in animal gut microbiomes

**DOI:** 10.1080/19490976.2026.2645267

**Published:** 2026-03-17

**Authors:** Jerry Elorm Akresi, Thi Van Thanh Do, Zhisong Cui, N. R. Siva Shanmugam, Sarah Moraïs, Itzhak Mizrahi, Edward A. Bayer, Jennifer M. Auchtung, Yanbin Yin

**Affiliations:** a Department of Food Science and Technology, Nebraska Food for Health Center, University of Nebraska – Lincoln, Lincoln, NE, USA; b Department of Life Sciences, Ben-Gurion University of the Negev, Be’er Sheva, Israel; c Department of Biomolecular Sciences, The Weizmann Institute of Science, Rehovot, Israel

**Keywords:** Mucin, cellulosome, mucinolysome, gut microbiome, CAZyme, *Limousia*, *Anaeromassilibacillus*

## Abstract

Mucins create a physical barrier that protects human and animal tissues from microbial pathogens. Here, we provide evidence that mucin degradation can be mediated by unique mucinolysomes, defined as extracellular cellulosome-like multi-enzyme complexes specializing in mucin degradation. We predicted the presence of mucinolysomes across 63 metagenome-assembled genomes (MAGs) and two isolated genomes of three anaerobic species *of Limousia*, including seven MAGs from human gut microbiome samples from six countries. We validated that mucins can support the growth of the *Limousia* strain ET540 as its sole carbon source, triggering the upregulation of most mucinolysome-related genes in ET540. We modeled the mucinolysome assembly by predicting cohesin‒dockerin interactions among most of the mucinolysome proteins using AlphaFold3. We performed metagenomic read mapping of 2897 fecal samples from various human cohorts and wild/domesticated animals against *Limousia* MAGs. We found that *Limousia* has a greater abundance and prevalence in farm animals than in humans. This study characterizes and adds the *Limousia* bacteria as unique member to the list of human and animal gut mucin glycan-degrading bacteria. Overall, we discovered that this novel gut bacteria genus (*Limousia*) uses a previously unrecognized molecular mechanism for highly organized mucin glycan degradation, shedding new light on microbe‒host interactions in the gastrointestinal tracts of diverse animal hosts, including humans.

## Introduction

Mucins are glycoproteins that form the mucus layers on epithelial cell surfaces, providing an important physical barrier that functions to separate the epithelium from potential threats, including those of gut microbes.^1^ While several mucin-associated gut microbes have been linked to a healthy intestinal barrier, excessive mucin glycan degradation by commensal and pathogenic bacteria can contribute to different gastrointestinal (GI) tract diseases in humans and animals.^2^ The human colonic mucus is mainly composed of the large mucin glycoprotein MUC2. MUC2 contains.^3^ These glycans are constructed from five different monosaccharides, L-fucose, *N*-acetyl-D-galactosamine, *N*-acetyl-D-glucosamine, D-galactose, and sialic acid (*N*-acetyl-neuraminic acid), which can be connected by more than a dozen different glycosidic linkages in mucins.^4^ Their degradation requires a broad repertoire of carbohydrate-active enzymes (CAZymes), spanning numerous glycoside hydrolase families, including GH2, GH20, GH29, GH33, GH84, and GH95, among others, encoded in many different.^5^ Mucins also contain non-sugar modifications, for example, with sulfate and acetyl groups, that can be removed by microbial activity. Reflecting this chemical complexity, a recent report described the extensive CAZyme arsenal from the mucinolytic bacterium, *Akkermansia muciniphila,* capable of complete degradation of O-linked mucin.^6^

Mucin-glycan degrading CAZymes are often clustered into polysaccharide utilization loci (PULs) for synergistic mucin degradation in the genomes of Gram-negative gut bacteria, such as *Bacteroides thetaiotaomicron.*^7–10^ Here, we report the first description of a mucinolysome, a novel cellulosome-like system comprising multiple CAZymes, uniquely encoded by a group of Gram-positive gut bacteria, to degrade mucin glycans in the GI tracts of farm animals and humans. Cellulosomes are multienzymatic complexes that were first discovered in 1983 in *Clostridium thermocellum* from hot springs, which are capable of efficient degradation of plant polymers, such as cellulose, hemicellulose and other plant cell wall polysaccharides.^11^^,^^12^ Cellulosomes contain hallmark protein domains, cohesins (Cohs) and dockerins (Docs), which are key structural modules that interact to form Lego-like multi-protein complexes.^13^ Cohesins are typically found as tandem repeats in scaffoldins, large structural proteins that generally lack catalytic functions. Dockerins, on the other hand, are present in CAZymes such as cellulases and hemicellulases. Unlike the PUL mechanism that controls the co-regulation of CAZyme genes as co-localized gene clusters,^7–10^ the cohesin–dockerin (Coh–Doc) interaction involves incorporation of the enzymes into the complex and cellulosome formation. Scaffoldins provide sites for the docking of multiple CAZymes to form highly organized swissknife-like multi-enzyme cellulosome complexes for the coordinated degradation of complex carbohydrates.^14^ More recently, the “amylosome”, a cellulosome-like system, was discovered in *Ruminococcus bromii* from the human gut for resistant starch degradation,^15^^,^^16^ suggesting that the cellulosome paradigm may also be extended to other complex carbohydrate degradation, such as mucin glycans.

To determine whether the cellulosome paradigm could be extended to mucin glycans, we extensively searched existing human and animal gut metagenomic databases and found a total of 65 gut bacterial genomes that may encode mucinolysomes. These genomes include 63 metagenome assembled genomes (MAGs) and two bacterial isolate genomes, which were isolated from chicken caecum.^17^ One of the isolated genomes (GCF_002160515.1, *Anaeromassilibacillus* sp. An172) was previously published in draft status,^17^ and the other (strain ET540, also known as kol109) was sequenced and assembled into a gapless complete genome in this study. The 63 MAGs included seven from humans in six different countries and 56 from farm animals. Using the genome taxonomy database (GTDB),^18^ we assigned all 65 genomes to a newly defined genus, *Limousia.*^19^ Using a single-copy core gene phylogeny, we classified all these 65 genomes into three distinct species. We experimentally verified that the *Limousia* strain ET540 grew well with mucin as the sole carbon source, and generated RNA-seq data comparing gene expression in defined medium with glucose (control) and mucin. We observed an upregulation of a majority of mucinolysome genes in ET540 grown on mucins. We also computationally predicted Coh–Doc interactions in the mucinolysome system using AlphaFold3. Finally, following our previous metagenomic read mapping approach,^20^ we investigated the relative abundance and prevalence of these mucinolysome-encoding *Limousia* species in humans and farm animals. We found that mucinolysome-encoding *Limousia* were detected at low abundances across different animal hosts, with a higher prevalence and abundance in farm animals than in human samples.

## Methods

### Search for mucinolysome-encoding MAGs in UHGG

**Figure 1.**
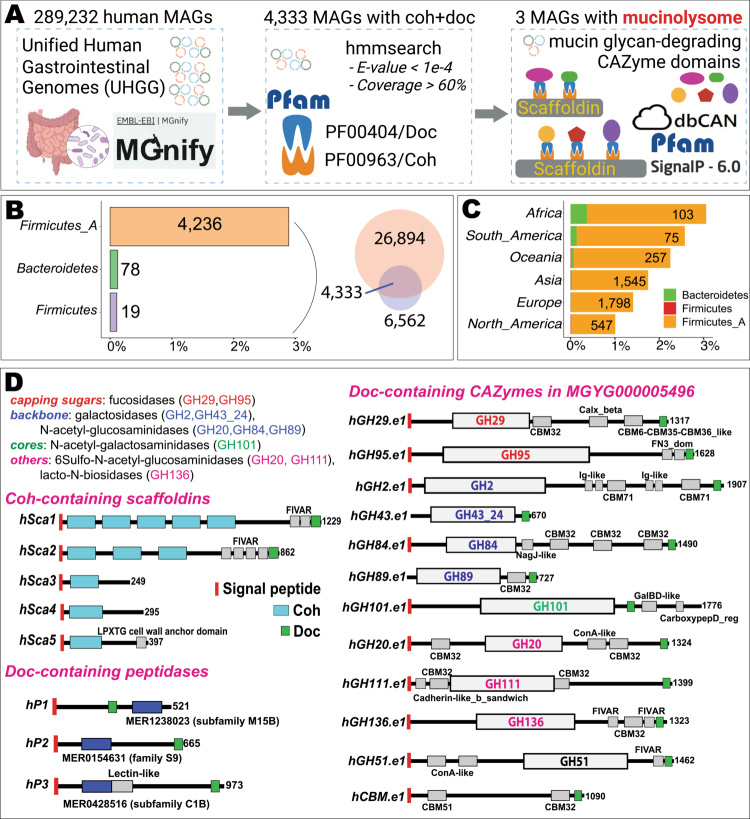
Discovery of mucinolysome-encoding MAGs in human gut microbiome. (A) Workflow to identify potential mucinolysomes in human gut MAGs. (B) Venn diagram shows the numbers of genomes enriched with Doc-containing proteins (26,894) and Coh-containing proteins (6562). The bar plot shows the bacterial phyla of the 4333 MAGs with Coh+Doc modules. The x-axis indicates the percentage of MAGs (# of MAGs with Coh+Doc/total # of MAGs in the specific phylum of UHGG). (C) The stacked bar plot shows the continents of the 4333 MAGs with Coh+Doc modules. The x-axis indicates the percentage of MAGs (# of MAGs with Coh+Doc/total # of MAGs in the specific continent of UHGG). (D) The dbCAN/Pfam domain diagrams of Doc-containing and Coh-containing proteins in one specific UHGG MAG: MGYG000005496 (Table 1). For Doc-containing proteins, only those with CAZyme and peptidase domains are shown. CAZyme domains are colored based on their biochemical activities from the literature^2^^,^^25^^,^^26^ and the CAZy website (cazy.org).^27^

**Figure 2.**
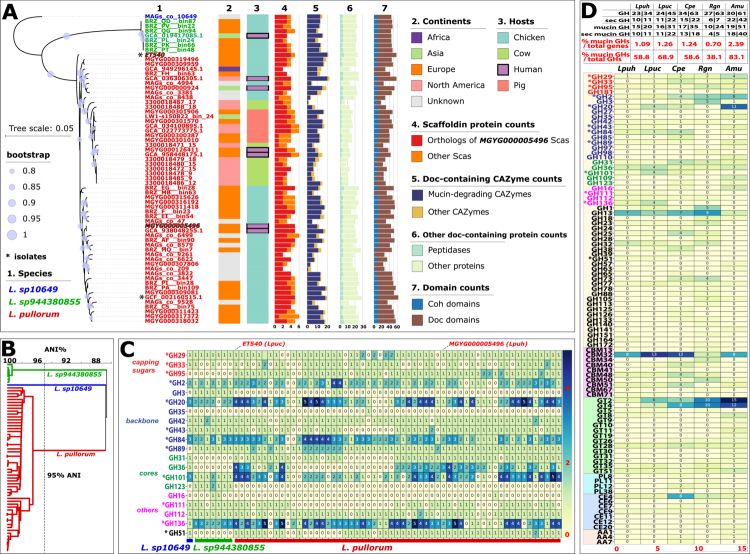
Sixty-five Limousia genomes and mucin-glycan-degrading CAZymes. (A) Phylogeny of the 65 genomes (see Tables S1 and S2 for more information) built from the single-copy core gene alignment. Two genomes are isolated genomes (GCF_002160515.1 and ET540, indicated with *), and the rest are MAGs. Only ET540 has a complete genome without gaps. All others are in contig or scaffold status. MGYG000005496 and ET540 are highlighted in italics and stroked font. Three species were identified according to this phylogeny. (B) Average nucleotide identity (ANI) dendrogram of the 65 genomes. (C) Heatmap of mucin glycan-degrading GH families (rows) in the 65 genomes (columns). The numbers are the protein counts of each family, irrespective of whether the proteins are Doc-containing or not. Families present in the ET540 mucinolysome (Figure 3) are indicated with *. (D) Heatmap of all CAZyme families (rows) in two Limousia genomes and three known mucin glycan-degrading genomes (columns). The numbers represent the protein counts of each family. Colored GH families (26 in total) are known for mucin glycan degradation according to the literature. The table on the top shows the family count and protein count separated by “|”. Counts for all GHs, secreted GHs (with signal peptides), mucin glycan GHs, and secreted mucin glycan GHs are listed (details in Table S3). The bottom two columns are the percentages of mucin glycan-degrading GHs calculated based on different denominators. The first one is calculated as the count of mucin-glycan-degrading GHs divided by the total gene count in the genome. The second one is calculated as the count of mucin glycan-degrading GHs divided by the total GH count in the genome. Lpuh: *Limousia pullorum* MGYG000005496, Lpuc: *Limousia pullorum* ET540, Cpe: *Clostridium perfringens* ATCC 13124, Rgn: *Ruminococcus gnavu*s ATCC 29149, Amu: *Akkermansia muciniphila* ATCC BAA 835.

We downloaded cohesin and dockerin hidden Markov models (HMMs) from Pfam V37.0. The length of the cohesin and dockerin profile HMMs were 139 and 58 residues, respectively.^21^^,^^22^ Using hmmsearch (http://hmmer.org/), we searched dockerin and cohesin HMMs against the Unified Human Gastrointestinal Genome (UHGG) database^23^ (Figure 1A). UHGG contains 289,232 genomes; most of them are MAGs, and 3.8% are isolated genomes. Among the 4333 MAGs that have both dockerin and cohesin modules, we searched for mucin-degrading CAZyme domains (Figure 2C) in the Doc-containing proteins by using dbCAN3^24^ with default parameters.

### Expand the search for mucinolysome-encoding MAGs in other sources

To expand our search for mucinolysome-encoding MAGs, the scaffoldins from the human MAG MGYG000005496^28^ were used as queries to search against the RefSeq database, with a total of 1,013,565 MAGs from various sources (Table S8, Figure S7A). The search was conducted using MMseqs2^29^ with sequence identity ≥50%, coverage ≥85%, and E-value <1e-5. MAGs with at least one scaffoldin match were retained and further searched for the co-presence of cohesin and dockerin modules, and for mucin-degrading CAZymes as described above.

To identify peptidases in the MAGs, DIAMOND was used (E-value <1e-10)^30^ to search against the MEROPS database.^31^ Finally, sulfatases in the MAGs were identified using hmmsearch (http://hmmer.org/) (E-value <1e-3) against the SulfAtlas database.^32^

### MAG taxonomic classification, quality check, and phylogenetic analysis

MAGs were classified taxonomically using GTDB-Tk (Genome Taxonomy Database, version 220).^33^ CheckM2 v.1.0.2^34^ was used to assess the MAG completeness and contamination levels. To infer species phylogeny based on MAGs, we used Panaroo^35^ to generate single-copy core gene alignments, FastTree^36^ for tree inference, and iTOL^37^ for visualization. Inter-MAG average nucleotide identity (ANI) was calculated by FastANI^38^ and ANI dendrogram was generated using Python scripts.

### MAG relative abundance and prevalence by read mapping of microbiome samples

For read mapping against MAGs in their original samples (Table S9), we used a pipeline (Figure S7B) consisting of bowtie2,^39^ SAMtools,^40^ InStrain quick_profile^41^ and CoverM (https://github.com/wwood/CoverM). The raw SRA files were quality-trimmed, and adapters and human contaminants were removed using bbduk from the BBTools suite,^42^ kraken^43^ and Seqtk (https://github.com/lh3/seqtk). The remaining reads were normalized to 10 million using SeqKit,^44^ and quality was assessed using FastQC.^45^

For read mapping against MAGs in 2897 fecal samples of different animal hosts (Table S10), we used the same pipeline as above (Figure S7B). From the read mapping results, the breadth of coverage (BC) and average read depth (ARD) were calculated for each MAG in each sample. The BC of a MAG was calculated as the count of bases that had at least one read mapped divided by the count of all bases in the MAG (i.e., the length of the MAG). The ARD of a MAG was calculated as the total length of all mapped reads divided by the length of the MAG. A MAG was considered present in a sample if it had a BC of ≥0.6 and ARD of ≥1. MAG relative abundance was calculated for each sample, and MAG prevalence was calculated across all samples. MAG relative abundance was calculated as the count of the mapped reads to the MAG divided by the count of all reads in the sample. MAG prevalence was calculated as the number of positive samples divided by the total number of samples.

Read mapping was also performed against 16S RNA genes and coding sequences (CDSs) of five scaffoldin proteins and 21 GHs of ET540. Proteinortho^46^ was run on all 65 MAGs using parameters “-cov = 90 -identity = 80 -sim = 1 -e = 1e-30” to find protein orthologs of CDSs. The presence, BC and ARD of the CDSs were determined in the same way as MAGs. 16S RNAs were predicted in 65 MAGs with Barrnap (https://github.com/tseemann/barrnap/tree/master). A 16S RNA was considered present in a sample if it had a BC of ≥0.6, ARD of ≥1 and read alignment sequence identity >99% (inStrain_profile: --min_read_ani = 0.99). These thresholds are more stringent than those used in a recent study^20^ to minimize false matches.

### Protein structure-based protein–protein interactions

We first extended all Pfam-predicted dockerin and cohesin domain regions to 10 amino acids on both sides. We then extracted these extended sequences for structure prediction. For sequences of each Coh–Doc pair, we ran AlphaFold3 (AF3)^47^ to model their complex structure and predict the protein-protein interaction (PPI) interface. AF3 produced various scores to measure the quality of the predicted structure and PPI, such as pTM (predicted template modeling score), ipTM (interface-predicted TM-score), IF_contacts (number of interface contacts), and IF_pLDDT (average local distance difference test score), for the predicted interfacial residues. We further ran FoldDock^48^ to calculate a pDockQ (predicted docking model quality) score for each predicted Coh–Doc complex structure from AF3. The pDockQ score combines the IF_ pLDDT and IF_contacts scores. A pDockQ score >0.3 was used as the threshold to filter out Coh–Doc complex structures with low PPI prediction confidence. Using the experimentally characterized Coh–Doc pairs as the ground-truth data, we calculated the recall, precision, and F1-score for our AF3-predicted Coh–Doc PPIs.

### Growth media for *L. pullorum* ET540

The ET540 strain (also known as kol109 in https://probio.vri.cz/en/blast-2/) was routinely passaged on Columbia agar plates, which comprised Columbia broth (Neogen, Lansing, MI, USA) solidified with 1.5% w/v agar (Franklin Lakes, NJ, USA). Mucin-dependent growth was characterized in defined basal medium (DBM, Table S6) or defined basal medium supplemented with 0.025% sodium sulfide and 0.2% tryptone (DBM #2) with no added carbon source, porcine gastric mucin (Type II mucin, Sigma Aldrich, St. Louis, MO, USA), or glucose added at the concentrations indicated in the figures. DBM agar plates were prepared by adding agar (Bacto, Thermo Scientific) to 1.5% w/v. All growth experiments were performed at 37 °C in an anaerobic chamber (Coy Laboratories, Grass Lake, MI, USA) with an atmosphere of 5% H_2_, 5% CO_2_, and 90% N_2_. All media were pre-reduced in the anaerobic chamber for >24 hr.

### Initial validation of *L. pullorum* ET540

The ET540 strain was obtained from Dr. Ivan Rychlík (Veterinary Research Institute, Brno, Czech Republic). The glycerol stock of strain ET540 was streaked on Columbia agar plates and incubated at 37 °C in an anaerobic chamber (gas atmosphere of 5% H_2_/5% CO_2_/90% N_2_) for 6‒7 d to obtain colonies with distinct morphology. Colonies were re-passaged on Columbia agar in an anaerobic chamber to ensure purity, DNA was isolated for validation of strain identity by whole-genome sequencing (details below), and new stocks in Columbia broth and preserved with 20% glycerol were created. For subsequent experiments, *L. pullorum* ET540 was passaged from a frozen stock onto Columbia agar for 6‒7 d prior to use.

### DNA sequencing and analysis of *L. pullorum* ET540

Full-genome sequencing was performed by Plasmidsaurus using a combination of long-read sequencing with Oxford Nanopore and short-read Illumina sequencing. Long-read sequencing was performed on PromethION P24 with an R10.4.1 flow cell; libraries were prepared with the rapid barcoding kit 96 V14. The basecalling model was ont-doradod-for-promethion on super-accurate mode. Read quality was set for a minimum Q-score of 10, with adapters trimmed by MinKnow. The genomes were assembled by removing 5% of the FASTQ reads with the lowest quality scores with Filtlong v0.2.1 (https://github.com/rrwick/Filtlong), followed by downsampling the reads to 250 MB via Filtlong to create a rough assembly with Miniasm v0.3.^49^ The reads were then re-downsampled to ~100X coverage, with a heavy weight applied to remove low-quality reads. High-quality ONT reads were assembled with Flye v2.9.1^50^ and polished with the reads generated during downsampling to ~100X coverage using Medaka v1.8.0 (https://github.com/nanoporetech/medaka). Genes were annotated with Bakta v1.6.1,^51^ contigs were analyzed with Bandage v0.8.1,^52^ genome completeness and contamination were analyzed with CheckM v1.2.2,^53^ and species/plasmids were identified through a combination of Mash v2.3^54^ against RefSeq genomes+plasmids, Sourmash v4.6.1^55^ against GenBank, and CheckM v1.2.2. Short-read Illumina sequencing was performed on a NextSeq12000 using a 2X150 bp read kit; libraries were prepared using SeqWell ExpressPlex 96 library prep kit. BWA^56^ was used to align raw Illumina FASTQ reads to the ONT-only assembly (R1 and R2 were aligned separately). BWA mem was used to align short reads to remove low-quality sequences from the ends of the reads. Polypolish v0.6.0^57^ was used to generate the final polished FASTA sequence, which was then annotated with Bakta using default parameters.

### Characterization of mucin-dependent growth of *L. pullorum* ET540

To initially characterize mucin-dependent growth by ET540, colonies grown on Columbia agar plates were resuspended in phosphate-buffered saline (PBS), serially diluted in PBS to 10^−5^ dilution, and 100  μL of each dilution was spread on agar plates without an added carbon source or with 0.2% w/v mucin under anaerobic conditions. The plates were incubated anaerobically at 37 °C for 5–6 d, after which the number of colony-forming units (CFU) on the plate with >50 colonies and <250 colonies was enumerated to determine the CFU/mL; based on the dilutions, the limit of detection was <10 CFU/mL. Images of the plates were captured using Syngene NuGenius, and all the images were adjusted to the same brightness and contrast in Adobe Photoshop prior to being embedded in Adobe Illustrator.

The maximum cell density (OD_620_) and growth rate were determined for cultures grown in DBM or DBM #2 broth with added glucose or mucin. Colonies grown on Columbia agar were used to inoculate DBM + 0.2% mucin broth cultures, which were allowed to grow anaerobically at 37 °C for 24‒48 h. The cultures were then diluted back to 5% v/v into broth with no carbon source or with different concentrations of mucin or glucose in 96-well culture plates. Growth was monitored over time by measurement of OD_620_ in a Tecan Sunrise plate reader (Tecan, Männedorf, Switzerland), with measurements collected every 20 min for 36–72 h. The OD_620_ data for each replicate well (*n ≥* 3/condition) were normalized to the starting OD_620_. The growth rate (*μ*) was determined by linear regression of natural log-transformed OD_620_ data during exponential growth (2–24 h). Doubling times were calculated from growth rates (ln(2)/μ). The data were plotted in GraphPad Prism v10.5.0.

### Comparison of *L. pullorum* ET540 growth to non-mucin- degrading species

To further demonstrate that *L. pullorum* ET540 growth was dependent on mucin, we compared the growth of ET540 to three species of gut microbes previously characterized as lacking the ability to degrade mucins^25^: *Clostridium butyricum* ATCC 19398, *Clostridium sporogenes* ATCC 3584, and *Lactobacillus johnsonii* NC533. For routine passage, *C. butyricum* and *C. sporogenes* were grown anaerobically at 37 °C in BHIS (Brain Heart Infusion [BD Difco, Franklin Lakes, NJ, USA] supplemented with 5 g/L yeast extract [Gibco Bacto, Thermo Fisher Scientific]) broth or 1.5% w/v agar, *L. johnsonii* was grown anaerobically at 37 °C in MRS Lactobacilli (BD Difco) broth or agar (1.5% w/v), and ET540 was grown anaerobically at 37 °C in DBM + 0.2% mucin.

To be consistent with previously published work,^25^ we assessed growth using Type III porcine gastric mucin (Sigma–Aldrich, St. Louis, MO, USA) that was dialyzed to remove free monosaccharides, disaccariches, and small oligosacchardies rather than Type II porcine gastric mucin. To facilitate comparisons across experiments, ET540 growth was assessed in Type II porcine gastric mucin in parallel. Specifically, Type III porcine gastric mucin was resuspended at 5 mg/mL in sterile distilled water, dialyzed in sterile distilled water with Specra/Por 1 standard RC tubing (6–8 kDa MWCO; Repligen, Boston MA USA) for 48  h, and lyophilized for 72  h in a Labconco Freezone 6 (Labconco, Kansas City, MO, USA). Mucin was then added to DBM at a final concentration of 2 g/L (0.2%) and pre-reduced in an anaerobic chamber for ≥ 24 h. *C. butyricum, C. sporogenes*, and *L. johnsonii* were cultured from frozen stocks onto BHIS (*C. butyricum* and *C. sporogenes*) or MRS (*L. johnsonii*) agar and grown anaerobically at 37 °C for 24 h. A total of 5–10 colonies were then used to inoculate BHIS or MRS broth, which was grown anaerobically at 37 °C for 16 h. *L. pullorum* was passaged on Columbia broth agar and DBM with 0.2% mucin (Type II porcine gastric mucin) as described above. Broth cultures were diluted in triplicate to 5% v/v in DBM with 0.2% Type III porcine gastric mucin and 1.8% glucose (~100 mM; concentration used in,^25^ or 0.2% Type II porcine gastric mucin (ET540 only), and anaerobic growth (OD620) at 37 °C was measured every 20 min for 48 h in a Tecan plate reader with OD620 values normalized to the starting OD620.

### Growth of ET540 bacterial strain for RNA sequencing

To evaluate the transcriptomic responses of ET540 in response to mucin or glucose as a carbon sources, triplicate cultures were grown in 40  mL of DBM #2 with 0.2% mucin for 48  h. Cultures were then diluted to 5% v/v into either 100 mL DBM #2 with 0.2% mucin or 0.2% glucose in triplicate for collection of samples for RNA extraction. In parallel, the inoculum was diluted back to 5% v/v into 96-well plates containing the same growth media, and growth was monitored in a Tecan Sunrise plate reader as described above. Samples (50 mL) were collected for RNA extraction after 12 h of growth (late exponential phase based upon growth of parallel cultures grown in a plate reader), pelleted by centrifugation at 4800 × g for 15 min at 15 °C, decanted and resuspended in 1 mL of TRIzol reagent (Invitrogen, Waltham, MA, USA); samples were stored at –80 °C until extraction.

### RNA extraction

RNA was extracted and purified using the TRIzol extraction protocol with Qiagen RNeasy. Specifically, 0.2  mL of chloroform per 1  mL of TRIzol reagent was added. The tubes were vigorously shaken for 15 s, incubated at room temperature for 2–3 min, and centrifuged at 12,000 × g for 15 min at 4 °C. The upper aqueous phase (approximately 60% of the original TRIzol reagent volume) was carefully transferred to a fresh RNase-free tube, and an equal volume of 75% ethanol was added and mixed gently. The RNA‒ethanol mixture was transferred onto an RNeasy spin column and centrifuged at ≥8000 × g for 15 s at 4 °C. Subsequent steps were performed according to the manufacturer’s protocol, with elution in a total of 80  μL of RNAse-free water (two-step elution with 50 and 30  μL).

The extracted RNA was quantified using a NanoDrop spectrophotometer (Thermo Fisher Scientific), assessing concentration, total RNA yield, and purity (A260/A280 and A260/A230 ratios). RNA integrity was assessed with a 4200 Agilent TapeStation following the manufacturer’s protocols, ensuring that the high-quality standards required for RNA sequencing were met. The RNA samples were aliquoted into RNase-free tubes and stored at –80 °C until shipment to the sequencing facility (Novogene, Sacramento, CA, USA).

### RNA-seq differential gene expression analysis

Paired-read FASTQ files from two groups, control group G and mucin group M (each with 3 replicates), were analyzed for differential gene expression. The read files were quality assessed using FastQC,^45^ aggregated using MultiQC^58^ and quality cleaned with trimmomatic.^59^ Post trimmed files were quality assessed again with FastQC. Using the reference-based method, HISAT2^60^ and SAMtools^40^ were used to align the clean reads against the ET540 reference genome and generate BAM files. We used gffreads^61^ to convert the ET540 genome to the “.gtf” format. The featureCounts program^62^ was then used for the quantification step to count the reads. Using the DESeq2,^63^ differentially expressed genes between the control group (G) and the mucin group (M) were identified using an adjusted *p*-value (FDR) threshold of <0.05 and an absolute log2-fold change (∣log2FC∣) threshold of >1 (2-fold change). KEGG^64^ pathway enrichment analysis was performed using clusterProfiler v4.12.6.^65^

## Results

### Seven human MAGs are identified to encode mucinolysomes

The co-presence of dockerin and cohesin modules in the same genome signifies the existence of cellulosome-like protein complexes in a bacterium. From the 289,232 genomes of the Unified Human Gastrointestinal Genome (UHGG) database^23^ (Figure 1A), we identified 4333 (1.5%) MAGs that encode both dockerins and cohesins (Figure 1B). As expected, most of these MAGs are from the Firmicutes phylum, and the remaining 78 (1.8% of 4333) are from Bacteroidetes. The surprising co-presence of dockerins and cohesins in MAGs of Bacteroidetes needs further investigation in the future, as no cellulosome systems have been described outside of the Firmicutes phylum. Interestingly, human populations with a higher intake of fiber-rich diets, according to the Global Dietary Database,^66^ have higher percentages of MAGs with Coh+Doc modules (Figure 1C, e.g., Africa, South America), suggesting that fiber-rich diets select cellulosomal bacteria.

At least 18 CAZyme families have been experimentally characterized in different gut bacteria for mucin glycan degradation^2^^,^^25^^,^^26^ (Table 1, Figure 1A). In the present study, three out of the 4333 UHGG MAGs were considered to possess mucinolysomes, as they encoded both (i) scaffoldins bearing multiple Coh domains and (ii) mucin-glycan-degrading CAZymes with Doc domains. One of the three MAGs (MGYG000005496, Figure 1D) has five scaffoldins (two with multiple Coh domains), 12 Doc-containing CAZymes, and three Doc-containing peptidases. Eleven of the 12 Doc-containing CAZymes in this MAG contain glycoside hydrolase (GH) domains. All but one of these mucinolysome proteins contain signal peptides, suggesting that they are secreted, which is consistent with cellulosomal systems.

**Table 1.** Seven human MAGs with mucinolysome systems.

MAG ID (source DB)	SRA ID	Country	# of contigs	Genome length (kb)	Total # of proteins	# of Coh proteins	# of Doc proteins (*)	Disease
MGYG000005496 (EBI MGnify)	ERR321631	Denmark	93	2,022,232	1827	5	10	Obesity
MGYG000126411 (EBI MGnify)	ERR209841; ERR209842; ERR210693; ERR210694	Spain	394	1,611,060	1463	5	8	Healthy
MGYG000000924 (EBI MGnify)	ERR1620320	China	57	2,081,481	1863	4	15	Crohn's disease
GCA_036306305.1 (NCBI GenBank)	SRR24600132; SRR24599998; SRR24600064; SRR24600036	USA	73	2,139,278	1896	4	20	NA
GCA_019417085.1 (NCBI GenBank)	DRR171913	Japan	367	1,914,663	1811	2	15	NA
GCA_938048255.1 (NCBI GenBank)	ERR321631	Denmark	96	2,057,936	1858	5	16	Obesity
GCA_958448175.1 (NCBI GenBank)	ERR9578163	Germany	114	1,969,338	1892	6	11	Alzheimer’s disease

^*^ Doc-containing proteins are predicted to have mucin-targeting GH domains.^1^

Ten GH families (Figure 1D) in the MGYG000005496 mucinolysome have known biochemical activities in cleaving the capping sugars: fucosidases (GH29, GH95), breaking down glycan backbones: galactosidases (GH2, GH43_24), *N*-acetyl-glucosaminidases (GH20, GH84, GH89), degrading glycan cores: *N*-acetyl-galactosaminidases (GH101), and breaking down other major components in mucins: 6-Sulfo-*N*-acetyl-glucosaminidases (GH20, GH111) and lacto-*N*-biosidases (GH136). The GH43_24 family has been recently characterized as a *β*-galactosidase in *Akkermansia muciniphila* for mucin degradation.^6^ GH51 and GH111 have not been previously shown to degrade mucins. However, GH51 was found in a PUL in the human gut *B. thetaiotaomicron* (BT3092–BT3103). This PUL contains GH2, GH43, and multiple sulfatases,^67^ suggesting that the GH51 in this PUL may also function in mucin glycan degradation. GH111, a keratan sulfate hydrolase family, contains an experimentally characterized 6-sulfo-*N*-acetyl-glucosaminidase.^68^ Keratan sulfate has not been found in gut epithelial tissues, but is structurally similar to mucins.^69^

To expand our search for mucinolysome MAGs, we further searched the five scaffoldin sequences present in the three MAGs against all human gut bacterial genomes in the RefSeq database. Using this approach, we identified four additional human MAGs (Table 1) in GenBank that may also encode mucinolysomes. One of them (GCA_938048255) appears to be derived from the same metagenome sample (ERR321631) as the UHGG MGYG000005496; however, they differ in the number of contigs, genome length, and number of proteins (Table 1). Overall, the original microbiome samples of the seven human MAGs were sourced from both diseased and healthy human individuals from six different countries in Europe, Asia, and North America. These findings suggest that these mucinolysome-encoding gut bacteria are widely distributed in different human populations and are unlikely to be contaminants from other sources. Although, the living environments or lifestyles of these humans are unknown, one MAG (GCA_036306305.1) was published in a recent study where the microbiomes of farmers and non-farmers were compared;^70^ this specific MAG was indicated from a non-farmer (NCBI biosample ID: SAMN38021763).

### Two bacterial isolate genomes and 56 MAGs of the genus *Limousia* from farm animals also encode mucinolysomes

As one of the five scaffoldins, hSca1 (Sca1 of human MAG MGYG000005496) is 1229 aa long with five Coh repeats (Figure 1D), we selected its sequence to search against the NCBI nr database and found that hSca1 has a very similar (98.94% sequence identity) protein hit (WP_087376890.1) in *Anaeromassilibacillus sp.* An172. The An172 genome (GCF_002160515.1) was sequenced from a pure bacterial culture isolated from a chicken caecum.^17^ Here, we sequenced ET540, a strain closely related to An172, which was also isolated from the chicken caecum. We generated the complete genome of ET540 with no gaps, annotated the two isolate genomes (ET540 and An172), and confirmed that they contained all the mucinolysome-related genes that the human MGYG000005496 possesses (Figure S1).

We further expanded the search for mucinolysomes to additional MAG databases. These databases included MAGs from humans living in different lifestyles,^70^^,^^71^ from different farm animals (cows,^28^^,^^72^ pigs,^28^^,^^73^^,^^74^ chickens,^75^^,^^76^ sheep,^77^ and from the Global Microbial Gene Catalog (GMGC).^74^ Using the same workflow (Figure 1A), we found 56 additional mucinolysome-encoding MAGs (Table S1) from different animal hosts, including 40 from chickens, seven from pigs, and nine from cows (Figure 2A, column 3). These MAGs were built from metagenomes sampled from Europe (France, Germany, Spain, Denmark, Czech Republic), North America (USA, Canada), Asia (China, Japan), and Africa (Ethiopia) (Figure 2A, column 2). All these MAGs contain 3–6 scaffoldins (with up to 12 Coh domains, Figure 2A, columns 4 and 7) and up to 22 Doc-containing CAZymes with mucin-degrading GH domains (Figure 2A, column 5). In addition, dockerins were also found in 31 other proteins with peptidase or other predicted functional domains (Table S2, Figure 2A, column 6). The An172 genome (GCF_002160515.1) encodes the most^55^ Doc domains.

In total, 65 genomes (63 MAGs and two isolate genomes, Figure 2A) were found to potentially encode mucinolysomes (Figure S1). All genomes were taxonomically annotated as *Anaeromassilibacillus* or *Acutalibacteraceae* sp. in their original databases. By running these genomes through GTDB v220,^18^ we classified all of them into a newly defined genus, *Limousia*, and most were assigned to the species *Candidatus Limousia pullorum.*^19^ A phylogenetic tree was built from the single-copy core protein sequence alignment of these 65 genomes (Figure 2A). We identified three distinct species, which were also supported by a genome average nucleotide identity (ANI) analysis using 95% as the threshold (Figure 2B). The phylogeny and ANI dendrogram also support that the *L. pullorum* genomes can be further divided into two subspecies, each of which contains an isolated genome (An172 and ET540). Following this new GTDB classification, we now renamed all these formerly known *Anaeromassilibacillus* genomes as *Limousia* genomes.

#### *L. pullorum* ET540 from chicken has a more complex mucinolysome system than MGYG000005496 from human and *Clostridium perfringens*

**Figure 3.**
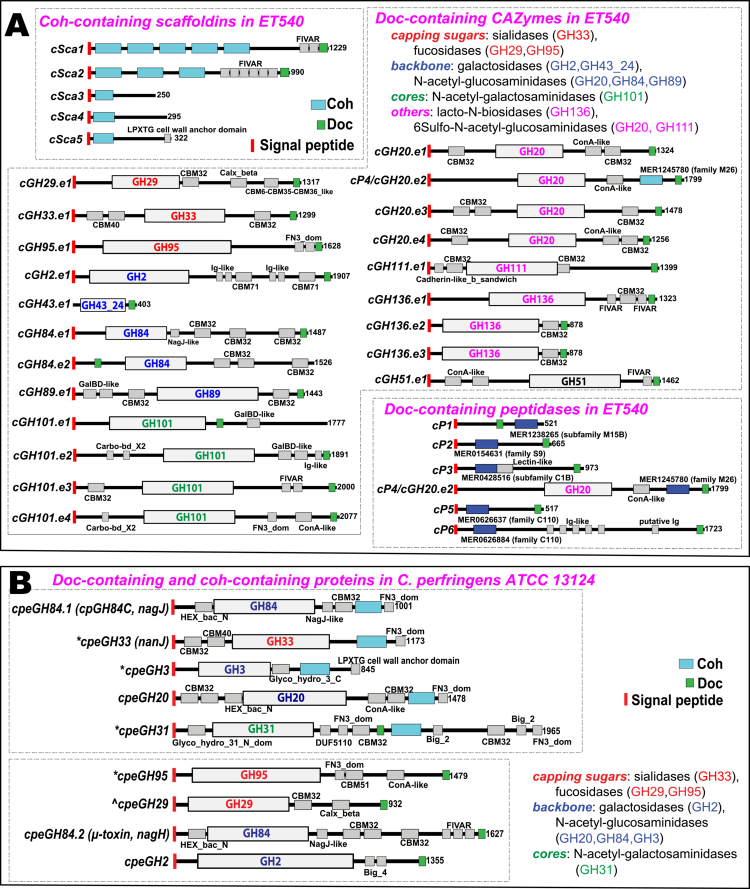
Comparison of domain diagrams of Doc-containing and Coh-containing proteins in two genomes. (A) ET540 and (B) ATCC 13124. For Doc-containing proteins, only those with CAZyme and peptidase domains are shown. CAZyme domains are colored based on their biochemical activities from the literature^2^^,^^25^^,^^26^ and the CAZy website (cazy.org).^27^ * Indicates that these proteins have a weak cohesin or dockerin signal that has failed to be identified by the Pfam cohesin and dockerin HMMs with E-value <1e-4 and coverage >60% (Figure 1A). ^ Indicates the GH29 protein that failed to be identified with the dockerin domain in the previous study.^78^

Compared to the human MAG MGYG000005496 (Figure 1D), the chicken ET540 genome has a more complex mucinolysome system (Figure 3A). ET540 encodes 21 Doc-containing CAZymes of 12 GH families, including a GH33 sialidase missing in MGYG000005496. ET540 has four GH101, four GH20, three GH136, and two GH84 proteins, compared to one copy of each in MGYG000005496. Interestingly, one of the GH20 proteins also contains a peptidase domain. Therefore, ET540 has a total of six Doc-containing peptidases (Figure 3A), compared to three in MGYG000005496. All scaffoldins and all Doc-containing peptidases comprise a signal peptide, similar to most Doc-containing CAZymes.

*Clostridium perfringens* ATCC 13124 has been previously shown to use Coh–Doc interactions to form multi-toxin complexes for animal tissue destruction.^78^ In this context, ATCC 13124 has five Coh-containing proteins, each having a single Coh module, previously known as X82.^78^ Interestingly, all five Coh-containing proteins also have a GH domain (Figure 3B), while no GH domains are present in the Sca proteins of ET540 (Figure 3A). Therefore, these Coh-containing proteins in ATCC 13124 are very different from traditional scaffoldins, which usually contain no catalytic domains but only multiple Coh tandem repeats. The only reported exception is scaffoldin CipV from *Acetivibrio cellulolyticus*^79^ that contains a GH9 domain in addition to seven Coh repeats. ATCC 13124 also has five Doc-containing GHs, including one GH29 protein that was overlooked in the previous study^78^ and one GH31 protein with both Coh and Doh domains. The five GH proteins include two known toxins in *C. perfringens*: a hyaluronidase (*μ*-toxin, NagH, GH84) and a sialidase (NanJ, GH33).^80^ Overall, ATCC 13124 has a very distinct and much simpler Coh–Doc interaction system (Figure 3B) than ET540.

ET540’s mucinolysome system has a total of 12 mucin glycan-degrading GH families (Figure 3, also indicated by “*” in Figure 2C). Eight other GH families have also been reported in the well-studied mucin glycan-degrading gut bacteria *A. muciniphila*, *C. perfringens*, *Bifidobacterium bifidum*, *R. gnavus,* and *Roseburia inulinivorans.*^2^^,^^6^^,^^25^^,^^26^ These eight GH families were found in *Limousia* CAZymes without Doc domains. For example, GH42 (*β*-galactosidases) and GH112 (lacto-*N*-biose phosphorylases) were present in most of the 65 *Limousia* genomes. Some families (e.g., GH3 *β*-*N*-acetylhexosaminidases and GH123 *β*-*N*-acetylgalactosaminidases) were almost exclusively found in one of the three *Limousia* species (sp944380855, Figure 2C), whereas other families (GH31 and GH36 *α*-*N*-acetylgalactosaminidases, GH95 and GH51 fucosidases) were almost exclusively absent in sp944380855. Overall, GH2, GH20, GH84, GH101, and GH136 tend to have multiple protein copies in each *Limousia* genome, while other families (e.g., GH29, GH33, GH42, GH43, GH89, GH31, GH112, and GH51) have only one copy in each genome (Figure 2C).

We further compared the total CAZyme repertories of the two representative *L. pullorum* genomes (MGYG000005496, or *Lpuh,* from humans, and ET540, or *Lpuc,* from chickens) against those of *A. muciniphila*, *C. perfringens* and *R. gnavus* (Figure 2D and Table S3). The two *Limousia* genomes encode 23 and 24 GH families for the degradation of glycans from different sources (34 and 45 GH proteins in *Lpuh* and *Lpuc*, respectively), which is lower than the number of GH families found in the three known mucin glycan-degrading genomes (*A. muciniphila*, *C. perfringens*, *R. gnavus*). However, when only considering the percentage of mucin glycan-degrading GHs (all rows in Figure 2C and all colored GH families in Figure 2D) over all genes in the genome, *Limousia* genomes have similar percentages (1.1% and 1.3%) as *C. perfringens* (1.2%), higher than *R. gnavus* (0.7%), but lower than *A. muciniphila* (2.4%). The percentage of mucin glycan-degrading GHs among all GHs was also highest in *A. muciniphila* (83.1%), followed by *Lpuc* (68.9%). The extracellular activities of GHs are important for mucin degradation.^81^ *A. muciniphila* had the highest percentages of GHs (68.85%) and mucin glycan GHs (78.43%) predicted to have a signal peptide, followed by *Lpuc* (48.89% and 70.97%) (Figure 2D, Table S3). Overall, 95.24% and 100% of the GHs containing signal peptides in *A. muciniphila* and *Lpuc* target mucins. Note that considering the Gram-negative *A. muciniphila* has inner and outer membranes, not all its GHs with signal peptides are secreted to the outside of the cell.

*A. muciniphila* has more copies of GH29, GH2, GH20, and GH16, and more unique families (GH181, GH27, GH35, GH97, GH110, and GH109) for mucin degradation (Figure 2D). The *Limousia* genomes also have unique families that are absent in the other genomes, such as GH136 and GH111. *Limousia* and *C. perfringens* are from the *Eubacteriales* order and share GH101, which is absent in the other two genomes, which are from the *Lachnospirales* order of Firmicutes (*R. gnavus*) and from the Verrucomicrobia phylum (*A. muciniphila*). *Limousia* and *C. perfringens* genomes also encode more CBM32, but fewer GT2 and GT4 proteins than the other genomes. All five genomes share GH13 and GH77 for *α*-glucan degradation (Figure 2D). *Limousia* genomes encode no PLs (polysaccharide lyases) and AAs (CAZyme of auxiliary activities), and very few CEs (carbohydrate esterases).

### AlphaFold3 predicts Coh–Doc interactions in the mucinolysome system of ET540

To better understand the potential for mucinolysome components to interact, we predicted the 3D structures for all cohesin and dockerin modules and their interactions in the mucinolysomes of *L. pullorum* ET540 and *C. perfringens* ATCC 13124 using AlphaFold3 (AF3).^47^ The predicted structures of Coh–Doc complexes were further processed by FoldDock^48^ to calculate pDockQ (predicted docking model quality) scores for all Coh–Doc pairs. The pDockQ score measures the confidence of the protein‒protein interaction (PPI) by considering the predicted local distance difference test (pLDDT) score (IF_pLDDT) and the number of residues in the PPI interface (IF_contacts). Previous reports showed that PPI models with pDockQ >0.23 are 70% likely correct^82^ and considered as acceptable.^48^

**Figure 4.**
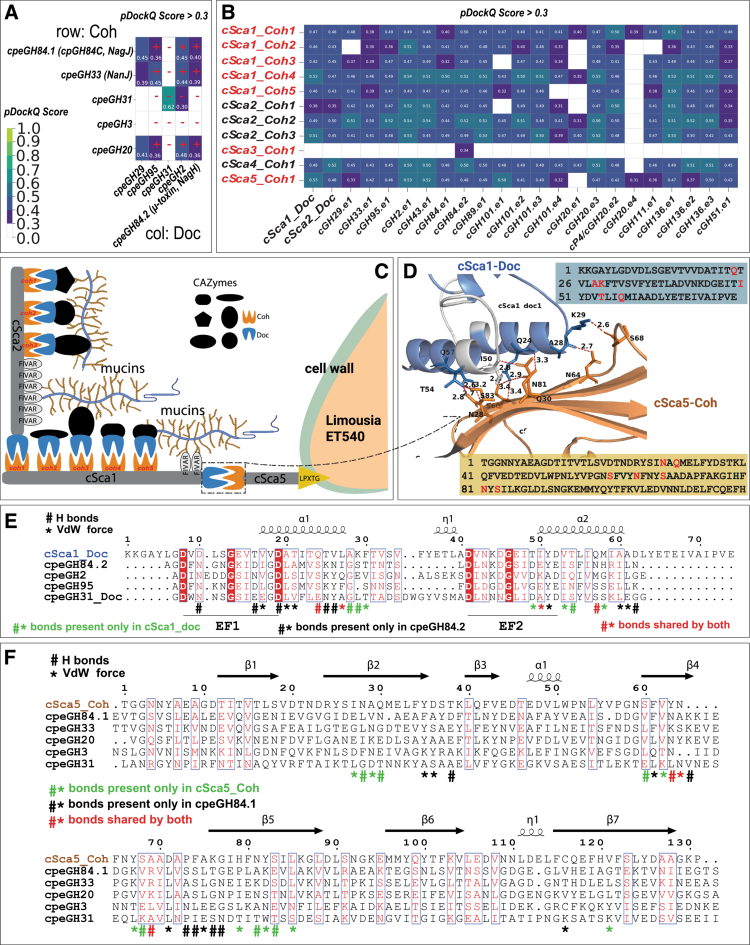
AlphaFold3 predicted functional Coh–Doc interactions. Dockerin and cohesin domain sequence pairs were used as input to AlphaFold3 for 3D structure and interaction predictions. The resulting PDB files were input to FoldDock to calculate the pDockQ scores. Scores greater than 0.3 are shown. (A) Protein‒protein interactions (PPIs) are predicted between cohesins (rows) and dockerins (columns) in the GHs of C. perfringens ATCC 13124. “+” and “−“ indicate the presence and absence of experimentally characterized PPIs.^78^ (B) PPIs are predicted between cohesins in scaffoldins (rows) and dockerins in GHs (columns) of ET540. (C) A simplified conceptual model is proposed to illustrate potential protein organization in the mucinolysome of ET540. (D) Predicted PPI interface between cSca1-Doc and cSca5-Coh. Hydrogen-bond contacts are shown as red dashed lines with corresponding distance <3.5 Å between atoms. The sequences of cSca1-Doc and cSca5-Coh are shown beside the PPI interface structure with residues highlighted corresponding to residues in the hydrogen-bond contact interface. (E) Sequence alignment of cSca1-Doc against dockerin sequences in four ATCC 13124 proteins. Key residues involved in hydrogen-bonding (#) and van der Waals (*) contacts are indicated. Residues for cSca1-Doc were predicted, while residues for ATCC 13124 proteins were published previously.^78^ The two EF-hand motifs are indicated. (F) Sequence alignment of cSca5-Coh against cohesin sequences in five published ATCC 13124 proteins.^78^

We first tested the feasibility of this approach on ATCC 13124 as a proof of concept. Previously, Coh–Doc interactions were experimentally detected in ATCC 13124,^78^ which revealed that the Coh-containing GH84.1 (NagJ), GH33 (NanJ), and GH20 proteins possessed strong binding properties to the Doc-containing GH84.2 (NagH), GH95, and GH2. The other two Coh-containing proteins (GH31 and GH3) exhibited no binding to Doc-containing proteins. With a pDockQ >0.3 threshold, AF3 predicted all these experimentally validated PPIs in ATCC 13124 (Figure 4A). Additionally, AF3 predicted PPIs between GH31-Coh and two dockerins: GH31-Doc and GH2-Doc. The interfaces of these two PPIs are ~18% disordered (Table S4); therefore, the PPIs might indeed exist but failed to be detected using an affinity ELISA-based assay.^78^ For the newly identified GH29-doc, AF3 predicted interactions with GH84.1 (NagJ)-Coh, GH33 (NanJ)-Coh and GH20-Coh. Therefore, AF3 predicted Coh–Doc PPIs with pDockQ >0.3 achieved an F1-score = 0.9 (recall = 1.0, precision = 0.82) in ATCC 13124.

We further applied this AF3-based method with the pDockQ >0.3 threshold on two additional gut bacterial species with experimentally detected Coh–Doc interactions. This method achieved an F1-score = 0.87 (recall = 1.0, precision = 0.76) in *Ruminococus bromii L2-63*^83^ for starch degradation (amylosome, Figure S8A) and F1-score = 0.68 (recall = 0.82, precision = 0.58) in *Ruminococcus champanellensis*^84^ for cellulose degradation (cellulosome, Figure S8B). Note that in *R. champanellensis*, some recombinant proteins with multiple Coh modules were used for affinity ELISA-based assay,^84^ which made it impossible to obtain reliable ground-truth data with only individual pairs of Coh–Doc PPIs. Therefore, the F1-score for *R. champanellensis* was underestimated.

Given the favorable performance of this AF3-based method on three different bacteria encoding different multi-enzyme systems via Coh–Doc PPIs, we applied this method with the pDockQ >0.3 threshold to predict Coh–Doc PPIs (Table S5) in ET540 (Figure 4B). Most Coh–Doc pairs were predicted to have interactions supported by higher pDockQ scores (compared to those in ATCC 13124), except for cSca3-Coh and cGH20.e4. Notably, cSca5 has a C-terminal LPxTG cell wall sortase anchoring domain, which is key for covalently linking the protein to peptidoglycans of bacterial cell walls.^85^ Interestingly, *N*-terminal cSca5-Coh was predicted to interact with both cSca1-Doc (pDockQ = 0.53, IF_pLDDT = 96.4, IF_contacts = 57) and cSca2-Doc (pDockQ = 0.48, IF_pLDDT = 94.7, IF_contacts = 53), as well as dockerins of most GHs. cSca1 and cSca2 both have multiple cohesin modules, which are predicted to interact with their own dockerins (i.e., cSca1-doc and cSca2-Doc) and those of most of the GHs. Thus, through Coh–Doc PPIs among these cSca proteins and GHs, multi-protein mucinolysome complexes of various components could form and be anchored onto bacterial cell walls through cSca5 (Figure 4C).

We further compared the predicted PPI interface of cSca5-Coh and cSca1-Doc with the experimentally determined PPI interface of cpeGH84.1-Coh and cpeGH84.2-Doc.^78^ Key residues in the two EF hand motifs (Figure 4D) were found to be conserved between ATCC 13124 and ET540, while those involved in the Coh–Doc interactions were not highly conserved but clustered in the sequence alignments (Figure 4E, F). This may reflect the species specificity in terms of Coh–Doc interactions to form the multi-protein complex.

### ET540 grows with mucin as the sole carbon source

**Figure 5.**
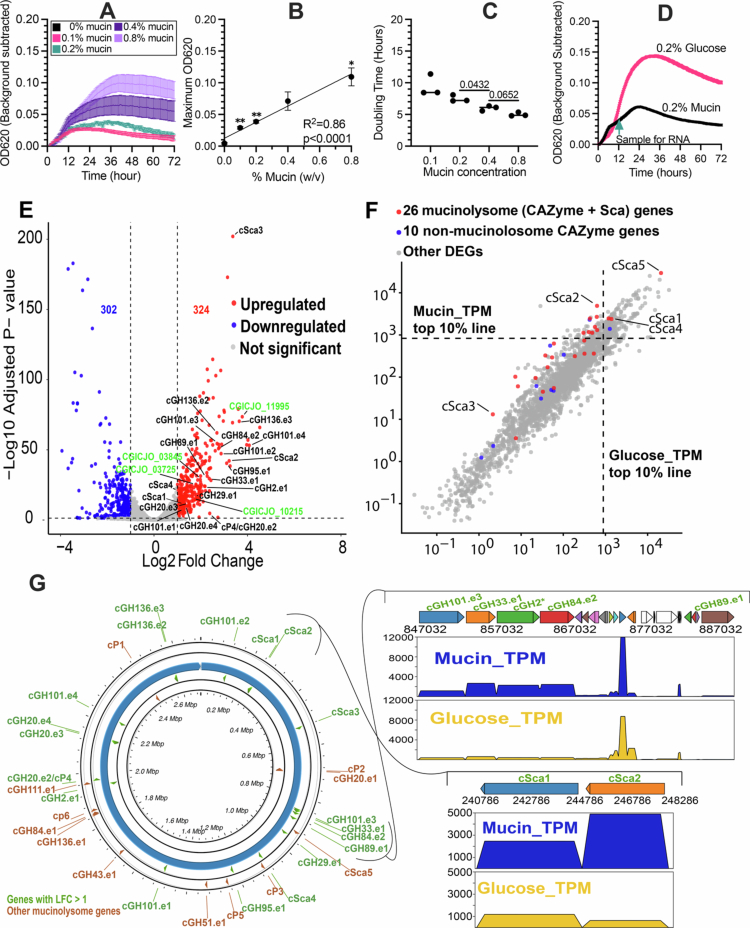
ET540 grows with mucin as the sole carbon source with most mucinolysome genes upregulated. In (A)–(C), ET540 was grown in DBM + 0.2% mucin for 48  h, diluted into fresh DBM medium containing the indicated mucin concentrations, where (A) growth over time in culture, (B) maximum OD_620_ relative to mucin concentration, and (C) doubling time from 2 to 24 h for each mucin concentration were plotted. In (A), the mean ± SEM values are plotted for triplicate cultures at each time point. In (B), mean ± SEM values are plotted for triplicate cultures at each mucin concentration. Linear regression was used to determine the goodness of fit (R^2^) and significance of slope deviation from zero, with *p* < 0.05 reported. The significance of differences in the maximum OD_620_ values at each mucin concentration relative to no mucin was determined by one-way ANOVA with Brown-Forsythe correction for unequal variances and Dunnett’s T3 correction for multiple comparisons, with ** indicating *p* < 0.01. In (C), each point represents a replicate, with the line indicating the median. The significance of the difference in doubling time between mucin concentrations was determined by one-way ANOVA with Brown–Forsythe correction for unequal variances and Dunnett’s T3 correction for multiple comparisons, with *p*-values < 0.1 reported. In (D), growth over time in culture was plotted as the mean ± SEM, and the time of sample collection for RNA extraction is indicated by a green arrow. (E) Differentially expressed genes (DEGs) between growth on glucose (DBM #2 + 0.2% glucose) and on mucin (DBM #2 + 0.2% mucin) are shown in the volcano plot. The horizontal line indicates the adjusted *p*-value (FDR) threshold of < 0.05. The vertical lines indicate the log2-fold change in log2FC >1 (two times higher in mucin, right line) or log2FC <–1 (two times lower in mucin, left line). Upregulated mucinolysome genes under growth on mucin are indicated (Table 2). Four non-mucinolysome DEGs are shown with green CGICJO gene labels. (F) Expression values in log10 scale of TPMs (transcripts per kilobase million) of all DEGs in mucin (y-axis) and gluclose (x-axis) samples. DEGs that are above or to the right of the top 10% of the cutoff lines are among the most highly expressed. (G) Circular view of the genomic locations of all mucinolysome CAZyme and peptidase genes. Those with log2FC >1 are shown in green. Read mapping coverage plots are shown for two gene clusters with upregulated mucinolysome genes. The y-axis shows the TPM values, and the x-axis shows the positions in the genome.

The ability of strain ET540 to grow with mucin as the sole carbon source was initially evaluated using defined basal medium (DBM, Table S6) on agar plates without an added carbon source or with 0.2% w/v porcine gastric mucin (Figure S2). From these experiments, we observed that mucin enhanced the growth of ET540 by more than 10^5^-fold, with no colonies recovered from DBM without an added carbon source (<10 CFU/mL). We further measured the growth of ET540 in DBM broth supplemented with different concentrations of mucin over time (Figure 5A). We observed a linear increase in maximal cell density (measured as OD_620_) as mucin concentrations increased (Figure 5B); similar results were observed in an independent experiment (Figure S3). We also observed modest decreases in doubling time during the exponential phase with increasing mucin concentrations (Figure 5C). In contrast, mucin-dependent growth was not observed in species (*Clostridium butyricum, C. sporogenes* and *Lactobacillus johnsonii*) previously shown^25^ to lack the ability to degrade mucins (Figure S4).

When ET540 was grown in DBM with added glucose, we observed that concentrations as high as 5% glucose did not support robust growth (Figure S4A, Figure S5A). Similar observations have been reported for *A. muciniphila*, where it was observed that supplementation with 0.2% tryptone would allow cells to metabolize glucose.^86^ We evaluated growth with glucose as a carbon source in DBM supplemented with 0.2% tryptone (DBM #2, Table S6) and observed glucose-dependent growth at 0.25%, 0.5%, and 1% glucose (Figure S5B), with similar maximum cell densities (Figure S5C) and doubling times (Figure S5C) at different concentrations.

**Figure 6.**
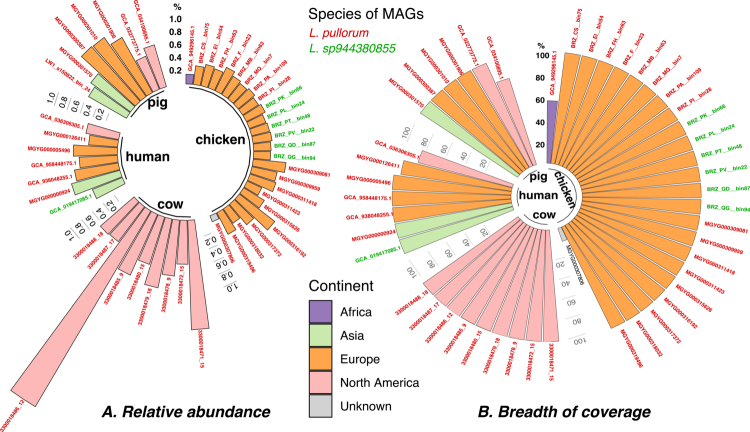
Relative abundance of Limousia MAGs determined by mapping the reads of the original samples. For each sample, all clean reads are mapped to the respective MAG using Bowtie (see Methods). The mapped reads are processed to calculate the relative abundance (RA) and the breadth of coverage (BC) for the MAG in the sample. (A) The RA of a MAG is calculated as the count of the mapped reads to the MAG divided by the count of all reads in the sample. (B) BC of a MAG is calculated as the count of bases that have at least one read mapped divided by the count of all bases in the MAG (i.e., the length of the MAG). The bars indicate the MAGs and are colored according to source continent. The bar labels are the MAG names that are colored based on the species defined in Figure 2A. The original read samples for 13 chicken MAGs were not located and were excluded in this analysis. Three MAGs (GCA_034100895.1, GCA_036306305.1, and MGYG000126411) that were assembled from multiple samples had their abundances in each of the samples calculated and then averaged.

To optimize growth conditions for comparing gene expression with mucin or glucose as the primary carbon source, we compared ET540 growth in DBM #2 with 0.1% or 0.2% mucin or glucose (Figure S6A), as ET540 did not grow in DBM with glucose. While we observed higher growth yields (Figure S6B) and shorter doubling times (Figure S6C) for cells grown in glucose compared to mucin, we observed that the cell densities were similar between ET540 in 0.2% mucin or 0.2% glucose after 12  h of growth (Figure 6D). We used these conditions for the growth of strains for RNA extraction and sequencing (described below) and collected growth measurements of replicate cultures grown in parallel (Figure S6D, E, F), with data from this replicate experiment exhibiting similar patterns of growth.

### Most mucinolysome genes are upregulated in ET540 grown on mucins

With the two growth conditions indicated in Figure 5D, we generated 11–14 Gb of short-read RNA-seq data per sample and mapped the clean reads to the ET540 genome. Using DESeq2,^63^ 626 (out of 2527) genes (Table S7) were identified as differentially expressed genes (DEGs) between the glucose group and the mucin group using an adjusted *p*-value (FDR) threshold of <0.05 and an absolute log2-fold change ∣log2FC∣ >1 (2-fold change). There were 324 upregulated DEGs and 302 downregulated DEGs in mucin compared to glucose (Figure 5E). The enriched KEGG functions of the upregulated DEGs included those associated with “glycosaminoglycan degradation”, “galactose metabolism” and “other glycan degradation”, which are all related to mucin degradation.

Interestingly, 19 (73.1%) of the 26 scaffoldin + Doc-containing CAZyme genes were upregulated in the presence of mucin (Table 2). Four of the five scaffoldin-encoding genes were DEGs (Figure 5E). The only exception is cSca5, which indeed has higher expression in mucin samples (FC = 1.69) but does not meet the log2FC>1 threshold (log2FC = 0.75, Table 2). Most interestingly, the cSca5-encoding gene exhibited the highest expression (Figure 5F) among all the 2527 ET540 genes (Table S7), with a TPM (transcripts per kilobase million) value = 29,423 in mucin samples; the cSca5-encoding gene also showed the second highest expression among all genes with a TPM = 20,802 in glucose samples (Figure 5F). The expression of the cSca5-encoding gene is six to seven times higher than that of the second most highly expressed mucinolysome gene (cSca2 in mucin samples and cSca4 in glucose samples, respectively, Table 2). This very high and constitutive gene expression of cSca5 (with a cell wall sortase anchoring motif) under both conditions strengthens its critical role in anchoring scaffoldin complexes to the bacterial cell wall and promoting the assembly of the mucinolysome and other potential degradative complexes (i.e., peptidases and other protein domains, Table S2).

Additionally, genes encoding six Doc-containing CAZymes (cGH84.e1, cGH111.e1, cGH136.e1, cGH20.e1, cGH43.e1, and cGH51.e1) and five Doc-containing peptidases (cP1, cP2, cP3, cP5, and cP6) were not upregulated DEGs (Table 2), but similar to the gene encoding cSca5, many of these genes presented increased expression in mucin samples but did not meet the log2FC>1 threshold. Interestingly, many mucinolysome genes form physically linked and co-expressed gene clusters (Figure 5G). For example, the genes encoding cSca1 and cSca2 are 287 base pairs apart and are co-upregulated in mucins. Three mucinolysome genes, encoding cGH101.e3, cGH33.e1, and GH84.e2, form a gene cluster with a non-mucinolysome GH2 gene (* in Figure 5G), with all four genes upregulated upon growth on mucins (Table S7). The gene encoding cGH89.e1 is located 17 genes downstream of this cluster, also upregulated upon growth on mucins (Table S7). Overall, the results of the RNA-seq experiment demonstrated that most mucinolysome genes were highly expressed and upregulated when ET540 was grown on mucins.

**Table 2.** Mucinolysic gene expression in ET540 sorted by TPM_M (transcripts per kilobase million when grown on mucin).

Gene ID	Gene name/CAZyme*	Mucinolysome	TPM_M	TPM_G	log2FC	Adj *P*-value	DEGs**
CGICJO_04010	cSca5	Yes	29423.84	20802.21	0.75	4.26E-08	
CGICJO_01195	cSca2	Yes	4901.69	631.74	3.22	7.88E-43	U
CGICJO_03730	cGH33.e1	Yes	2682.69	623.14	2.36	2.80E-30	U
CGICJO_01190	cSca1	Yes	2463.29	1187.68	1.31	7.24E-13	U
CGICJO_03740	cGH84.e2	Yes	2450.03	428.50	2.77	1.71E-54	U
CGICJO_04735	cSca4	Yes	2391.40	1390.71	1.03	1.48E-21	U
*CGICJO_03735*	*GH2*	No	*2294.58*	*421.43*	*2.7*	*3.62E-55*	*U*
CGICJO_04135	cGH29.e1	Yes	1553.41	563.54	1.72	3.78E-19	U
*CGICJO_02795*	*GH112*	No	*1392.12*	*1269.76*	*0.39*	*7.89E-03*	
CGICJO_06705	cGH101.e1	Yes	1305.38	635.12	1.30	1.62E-08	U
CGICJO_03725	cGH101.e3	Yes	1170.98	402.92	1.79	4.14E-26	U
CGICJO_09790	cGH20.e4	Yes	1125.86	485.76	1.47	3.95E-11	U
CGICJO_08830	cGH2.e1	Yes	1123.93	300.89	2.16	7.61E-25	U
CGICJO_09785	cGH20.e3	Yes	737.38	314.30	1.49	1.24E-12	U
CGICJO_11990	cGH136.e3	Yes	626.21	59.90	3.65	4.80E-71	U
*CGICJO_11995*	*GH136*	No	*557.68*	*48.74*	*3.78*	*2.82E-74*	*U*
CGICJO_08435	cGH84.e1	Yes	362.00	530.18	–0.30	0.016	
CGICJO_08900	cGH111.e1	Yes	359.99	318.36	0.43	0.003	
*CGICJO_03845*	*GH84*	No	*339.17*	*102.61*	*1.98*	*1.96E-29*	*U*
CGICJO_02305	cSca3	Yes	326.07	37.52	3.37	9.31E-203	U
CGICJO_08430	cGH136.e1	Yes	312.95	188.02	0.99	1.80E-12	
CGICJO_11985	cGH136.e2	Yes	293.16	54.27	2.69	8.90E-63	U
CGICJO_03840	cGH89.e1	Yes	171.60	42.38	2.28	5.31E-32	U
CGICJO_05460	cP5	Yes	167.63	264.65	–0.41	9.86E-04	
CGICJO_04790	cP3	Yes	116.88	162.26	–0.22	0.05	
CGICJO_11355	cP1	Yes	111.94	70.45	0.92	7.79E-12	
CGICJO_10190	cGH101.e4	Yes	102.19	7.28	4.08	6.73E-54	U
CGICJO_08925	cGH20.e2/cP4	Yes	95.11	21.99	2.38	2.03E-05	U
*CGICJO_10215*	*GH36*	No	*61.36*	*23.04*	*1.67*	*2.18E-16*	*U*
CGICJO_05315	cGH95.e1	Yes	60.52	8.40	3.11	3.66E-41	U
CGICJO_05805	cGH51.e1	Yes	55.09	59.69	0.14	0.48	
*CGICJO_06105*	*GH36*	No	*49.78*	*51.93*	*0.19*	*0.11*	
*CGICJO_04835*	*GH42*	No	*47.6*	*58.79*	*–0.05*	*0.67*	
CGICJO_03095	cGH20.e1	Yes	45.12	32.84	0.72	0.002	
CGICJO_03090	cP2	Yes	44.44	36.39	0.55	0.036	
*CGICJO_02140*	*GH31*	No	*31.02*	*29.66*	*0.32*	*0.026*	
CGICJO_00480	cGH101.e2	Yes	13.13	2.13	2.87	5.14E-48	U
CGICJO_08465	cP6	Yes	11.96	68.91	–2.28	2.43E-69	
CGICJO_07620	cGH43.e1	Yes	3.56	7.50	–0.82	4.24E-04	
*CGICJO_10290*	*GH36*	No	*2.33*	*2.19*	*0.34*	*0.33*	
*CGICJO_10285*	*GH36*	No	*1.24*	*1.14*	*0.36*	*0.45*	

^*^ U: upregulated in mucin (log2FC >1).

^**^ Non-mucinolysome genes are listed with encoded CAZyme domains instead of gene names.

In contrast to the 19 (73.1%) mucinolysome genes (encoding scaffoldins + Doc-containing CAZymes), only four (40%) of the 10 non-mucinolysome mucinolytic CAZyme genes were significantly upregulated (Table 2). Interestingly, three (CGICJO_03735|GH2, CGICJO_03845|GH84, CGICJO_11995|GH136) of the four upregulated non-mucinolysome genes were closely clustered in the gene neighborhood of the upregulated mucinolysome genes. For example, CGICJO_03735 (cGH2, * in Figure 5G) is clustered with cGH101.e3, cGH33.e1, and cGH84.e2. Additionally, the top 10% most highly expressed genes in mucin samples included 11 (42.3%) mucinolysome genes, compared to only 2 (20%) non-mucinolysome mucinolytic CAZyme genes (Figure 5F). Overall, the 26 mucinolysome genes exhibited substantially higher gene expressions (Figure S9A) and up-regulations (Figure S9B) than the 10 non-mucinolysome genes in the mucin samples. All these data strongly support that mucinolysome genes encode protein important for mucin utilization in ET540.

### Prevalence and abundance of mucinolysome-encoding *Limousia*

To study the abundance of these mucinolysome-encoding *Limousia* MAGs, we downloaded the original metagenomic reads used to construct these MAGs (if available) and mapped them back to these MAGs (see Methods). We found that the abundances of the seven human MAGs, their abundances were low (0.06%–0.61%) in their original samples (Figure 6A, Table 1). The abundances of MAGs from other animals, their abundances range between 0.0098% and 12.51% (Figure 6A). MAGs from cows had higher abundances, followed by MAGs sourced from pigs and humans. The chicken MAGs had the lowest abundances in their original samples. Despite their low abundance, most MAGs had almost 100% mapping breadth of coverage (Figure 6B), verifying their existence in the original samples. Overall, the mucinolysome-encoding *Limousia* bacteria have low abundance in different animal hosts (<1% for most MAGs).

**Figure 7.**
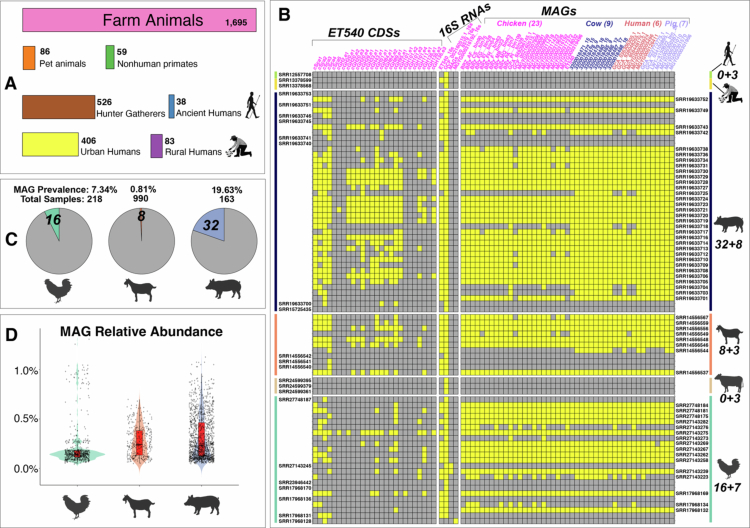
Prevalence and abundance of Limousia MAGs in different human and animal hosts. (A) The total fecal sample count was 2897. Sample counts from different animal and human hosts are shown as bars. (B) Heatmap of read mapping against MAGs, 16S RNAs, and ET540 mucinolysome CDSs as reference sequences. Yellow color indicates mapped or positive mapping, and gray color indicates unmapped mapping. The rows are sample SRA accessions, and the columns are references (MAGs/16S RNAs/CDSs). The rows are ordered by their hosts. SRAs shown on the right side are samples mapped to MAGs, and SRAs shown on the left side are samples unmapped to MAGs but mapped to 16S RNAs or CDSs. Two numbers are shown on the right side (e.g., 32+8): the first number is the count of samples (SRA IDs on the right) mapped to MAGs, and the second number is the count of samples (SRA IDs on the left) unmapped to MAGs but mapped to 16S RNAs or CDSs. Reference IDs are color-coded by host origin. (C) MAG prevalence (positive sample count divided by total sample count) in different hosts is shown as pie charts. (D) Boxplot of MAG relative abundance in positive samples grouped by hosts.

The prevalence of mucinolysome-encoding *Limousia* were studied by mapping metagenomic shotgun reads of 2897 fecal samples from various human cohorts and wild/domesticated animals (Figure 7A). To reduce the computational cost, we subsampled 10 million reads from each sample and mapped the clean reads to three different reference datasets (Figure 7B): (i) the entire genomes of 65 *Limousia* MAGs, (ii) 16S RNA sequences of four MAGs (no 16S RNAs were found in other MAGs), and (iii) coding sequences (CDSs) of five scaffoldin proteins and 21 GHs of ET540. Using inStrain^41^ and CoverM, we calculated the breadth of coverage (BC) and average read depth (ARD) for each MAG, 16S RNA, and CDS in each sample (see Methods).

As a result of the above-described approach, a total of 56 samples were found to be mapped to a total of 45 MAGs (Figure 7B). These 56 samples were therefore considered to encode the mucinolysome system. Note that cross-mapping could occur, meaning that read samples can be mapped to MAGs of different hosts. For example, the eight sheep samples were mapped to MAGs of chicken, cow, human, and pig meeting our thresholds (BC of ≥0.6 and ARD of ≥1). The prevalence of mucinolysome in different host samples was calculated as the number of positive samples divided by the total number of samples (Figure 7C). We observed the presence of MAGs in 16 chicken samples (prevalence rate = 7.34%), eight (0.81%) goat samples, and 32 (19.63%) pig samples. No samples from cow, human, non-human primate, and other animals were positive. This was unexpected since our 65 *Limousia* MAGs included seven MAGs from human and eight MAGs from cows (Figure 2A). Overall, a total prevalence rate of 1.94% was observed across all the samples. All 56 positive samples were sourced from China except for one from Germany. Notably, seven out of the 32 pig samples (Figure 7C) were derived from animals reported to have diarrhea, and the rest were from healthy piglets, but no disease information was found available for the chicken and goat samples.

In addition to MAGs, we also mapped the clean reads to the 16S RNA sequences of the four MAGs and the ET540 CDSs of the five scaffoldin proteins and 21 GHs. All 56 positive samples mapped to the MAGs were confirmed by positive mapping to at least one 16S RNA and one CDS (Figure 7B). Interestingly, we also found that 24 new samples mapped to at least one 16S RNA or one CDS. These 24 samples included three human samples and three cow samples, which were only mapped to 16S RNAs, meeting stringent thresholds: BC of ≥0.6, ARD of ≥1, and read alignment sequence identity >99%. Given the low abundance of mucinolysome-encoding *Limousia*, it is likely that these are true positive samples for the presence of mucinolysomes. The presence of mucinolysomes in pig, chicken, and goat was well supported despite the low prevalence (Figure 7C) and abundance (Figure 7D) in read mapping.

## Discussion

Mucins form a protective layer covering the epithelial surfaces in humans and animals. In the GI tract, mucin glycans are degraded by mutualistic microbes, such as *A. muciniphila* and *B. thetaiotamicron*, and pathobionts, such as *C. perfringens* and *Ruminococcus torques*. Mucin glycan-degrading commensal microbes can contribute to intestinal homeostasis through modulation of intestinal renewal and immune tolerance. For example, *A. muciniphila* stimulates intestinal stem cell proliferation and the differentiation of stem cells into Paneth and goblet cells,^87^ reduces intestinal damage after radiation or methotrexate treatment,^88^ and modulates the mucosal immune system, promoting the tolerance of mutualistic and commensal microbes.^89^ *C. perfringens* contributes to colonization by strains associated with antibiotic-associated diarrhea,^90^^,^^91^ whereas *R. torques* is enriched in patients with inflammatory bowel diseases.^92^^,^^93^ Thus, the discovery of new mucin-degrading microbes and molecular mechanisms has a potential major impact on biomedicine, particularly for understanding the balance between mucin glycan degradation that enhances barrier function and degradation that increases susceptibility to disease.

This study characterizes and adds *Limousia* bacteria as unique members to the list of human and animal gut mucin glycan-degrading bacteria.^25^ Consistent with this observation, 68.9% of the GHs in the *Limousia* ET540 genome are predicted to contribute to mucin glycan degradation, which is higher than both *C. perfringens* and *R. gnavus,* although lower than *A. muciniphila* (Figure 2D, Table S3). Furthermore, most enzymes involved in mucin glycan degradation in *Limousia* are likely associated with mucinolysomes, which are large extracellular multi-enzyme complexes formed through hallmark cellulosomal Coh–Doc interactions. This represents a new molecular mechanism for the processive degradation of mucin by mucin glycan-degrading CAZymes and peptidases (Figure 4C). This contrasts with other well-studied gut bacteria, such as *Bacteroides* spp., which use the polysaccharide utilization loci (PUL) mechanism for mucin utilization, where genes encoding CAZymes, transporters, and regulators form co-regulated gene clusters in genomes.^7^^,^^8^ *A. muciniphila* species also encode mucin utilization loci (MUL), which are composed of type IV-like pili proteins and transporters that may transport mucinolytic enzymes extracellularly or to the periplasm, and import mucin glycans and their partially degraded products into the cell to form the intracellular compartment called mucinosomes for further degradation.^81^ Thus, *Limousia* represents a new model microorganism to study the highly organized mucin glycan degradation using the novel mucinolysome mechanism.

The mucinolysome mechanism discovered in *Limousia* bacteria follows the classical cellulosome paradigm well described in *Clostridium* and *Ruminococcus* bacteria.^14^ Our study presents the first evidence that this cellulosome architecture can be utilized in animal glycan mucin degradation, in addition to the degradation of plant polysaccharides (lignocelluloses and starches). Although Adams et al. previously showed that *C. perfringens* ATCC 13124 uses Coh–Doc interactions to form complexes for the degradation of host-associated glycans,^78^ these complexes (Figure 3B) do not appear to follow the classical cellulosome architecture with the assembly of multiple Doc-containing CAZymes on scaffoldin proteins with multiple Coh-domains. Rather, this organization appears to facilitate pairwise interactions between GH proteins. Similarly, *A. muciniphila* encodes a GH31 protein with a single dockerin domain and a single cohesin domain, whereas *R. gnavus* lacks any dockerins or cohesins. In contrast, *Limousia* ET540 preserves the classical cellulosome architecture^14^ for mucin glycan degradation, as the five scaffoldins do not have GH domains, and tandem Coh repeats are present in cSca1 and cSca2 (Figure 3A). Future experimental studies are needed to fully characterize the differences between these approaches to mucin glycan degradation and evaluate the potential competitive growth advantages of these different mechanisms for mucin glycan degradation.

Structure-based prediction of Coh–Doc interactions, predicted using models validated by known interactions in three gut bacterial species (*C. perfringens* ATCC 13124,^78^ *R. bromii L2-63,*^83^ and *R. champanellensis),*^84^ were further applied to predict Coh–Doc interactions in *Limousia* ET540. The results revealed that most of the scaffoldins (except cSca3) could interact with all mucin glycan-degrading CAZymes and peptidases (Figure 4B). Interestingly, the longest cSca1 and cSca2, which contain five and three cohesin repeats, respectively, can potentially use their C-terminal dockerins to interact with the cell wall-anchoring cSca5. These interactions likely play a key role in the assembly of large multi-scaffoldin mucinolysome system and its attachment to bacterial cell walls. More interestingly, the gene encoding cell wall-anchoring cSca5 was the most highly expressed gene among all the genes in the genome (Table 2, Figure 5F, Table S7, ranking 1^st^ in mucin and 2^nd^ in glucose growth media), and was upregulated (1.7-fold) in mucin-containing growth media. The genes encoding the other four scaffoldins were all significantly upregulated (>2-fold) in mucin-containing growth media. While future experimental validations of these interactions are needed, our initial mucinolysome model (Figure 4C) likely underestimates the complexity of the real mucinolysome system in nature.

In addition to CAZymes, peptidases and sulfatases are also important for complete mucin degradation. The *Limousia* ET540 genome has a total of 92 peptidases according to a BLASTP search against the MEROPS database (E-value <1e-10), and nine sulfatases according to a hmmsearch against the SulfAtlas database (E-value <1e-3) (Table S7). Six of the peptidases contained dockerins (Figure 3A), and six peptidases were upregulated in ET540 grown in the presence of mucin. No sulfatases contained dockerins, and none were upregulated upon growth on mucin. It is known that very few bacterial sulfatases have been experimentally characterized and are thus less represented in the databases.^32^ Given that the ET540 isolate can grow well on mucins, novel sulfatases that do not share sequence similarity with known enzymes are likely to be discovered in *Limousia*.

Our comprehensive read mapping-based search revealed that the mucinolysome-encoding *Limousia* has low abundance in various human and animal gut microbiome samples. The seven human MAGs had relative abundances between 0.06% and 0.61% in their original samples (Figure 6A), which were lower than those reported for *R. gnavus* (average 0.67%),^94^ *A. muciniphila* (2.62%),^95^^,^^96^ and *C. perfringens* (average 1.16%)^95^ in healthy humans. The relative abundances of *Limousia* in other animals are also generally lower than 1% (Figures 6A and 7D). The prevalence of *Limousia* also exhibits large differences in different animals that have been studied: chicken (7.34%), goat (0.81%), and pig (19.63%). No other human microbiome samples beyond those listed in Table 1 were mapped to *Limousia* MAGs, while the reported prevalence of *R. gnavus* in healthy humans is very high (average 43.1%,^94^ as is *A. muciniphila* (47.1%)^95^^,^^96^ and *C. perfringens* (average 29.3%).^95^ Nevertheless, it is of note that *Limousia* 16S RNAs were mapped to three human samples (one ancient human and two rural humans) (Figure 7B). A search using the ET540 16S RNA gene to query the NCBI NT database revealed another 16S RNA match (FJ503849) from an intestinal mucosal biopsy of a Crohn's patient with 99.6% sequence identity. These findings suggest that, despite their very low abundance, the evidence is strong for the existence of mucinolysome-encoding *Limousia* in humans, as they are present in historically and geographically distant samples. Given their low abundance and prevalence, human *Limousia* bacteria might have been acquired from farm animals, which are among many sources of human microbiome transmissions.^97^

To summarize, our findings reveal a novel anaerobic gut bacteria genus (*Limousia*) using a previously unrecognized mechanism for mucin glycan degradation. Our study provides a framework to investigate how microbial activity shapes gut health, influences the balance between mucin glycan degradation and mucin turnover, and affects host–microbe interactions and nutrient dynamics across diverse animal species.

Finally, as a limitation of this study, the direct Coh–Doc bindings must be biochemically validated to prove the existence of mucinolysomes, which is beyond the scope of this genomics-focused study. In the future, we will use the ELISA method^98^ that has been widely used to characterize Coh–Doc bindings in cellulosomes and amylosomes. The preprint of this paper is available on bioRxiv.^99^

## Supplementary Material

TableS8.xlsx TableS8.xlsx

TableS1.xlsx TableS1.xlsx

TableS2.xlsx TableS2.xlsx

TableS6.xlsx TableS6.xlsx

TableS9.xlsx TableS9.xlsx

TableS3.xls TableS3.xls

TableS7.xlsx TableS7.xlsx

TableS4.xlsx TableS4.xlsx

TableS10.xlsx TableS10.xlsx

TableS5.xlsx TableS5.xlsx

Supplemental Material Supplementary_file.docx

## Data Availability

The raw sequencing reads of ET540 genome and the genome assembly are available at https://www.ncbi.nlm.nih.gov/bioproject/?term=PRJNA1339042. The faa, fna and gff files of all 65 mucinolysome-encoding *Limousia* genomes have been deposited in figshare at https://doi.org/10.6084/m9.figshare.30192742.

## Code availability statement

Code is freely available at https://github.com/elormakresi/Mucinolysome-in-gut-microbiomes-of-farm-animals-and-humans.
