# Supplementary material for: Limousia bacteria encode mucinolysome for mucin utilization in animal gut microbiomes
Source: Gut Microbes. 2026 Mar 17;18(1):2645267. doi: 10.1080/19490976.2026.2645267 (PMC12997984; doi:10.1080/19490976.2026.2645267)
Supplement: Supplemental Material — Supplementary_file.docx [file KGMI_A_2645267_SM7680.docx]

**Supplementary figures**


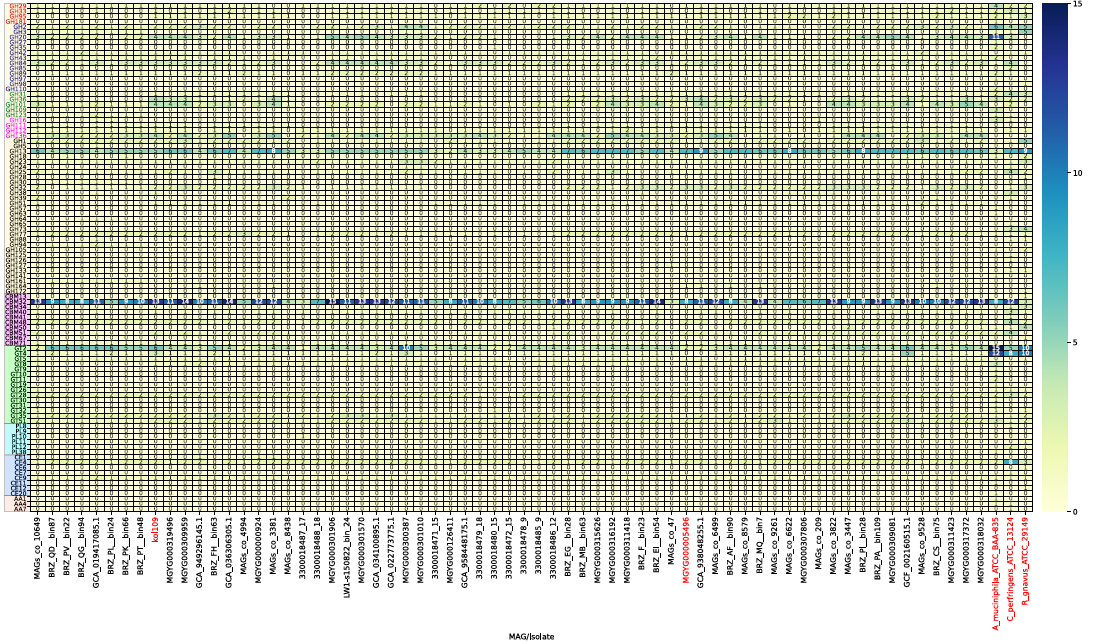


***Figure S1****: Heatmap of all CAZyme families (rows) in 65 Limousia genomes and three known mucin-degrading genomes (columns). The values indicate the number of genes per bacterium for the given protein family. Colored GH families are known for mucin degradation according to the literature. ET540 is also known as kol109.*

*
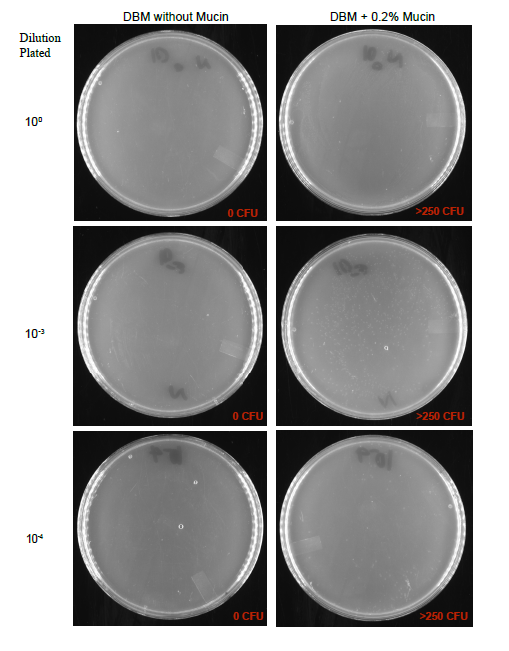
*

***Figure S2****: Limousia pullorum ET540 grows on DBM with 0.2% mucin as the sole carbon source. Growth of L. pullorum ET540 on DBM, without an added carbon source or with 0.2% porcine gastric mucin added as the sole carbon source, was assessed by measuring colonies formed from L. pullorum at different dilutions of inoculum. Colony forming units (CFU)/mL were calculated as described in methods, with DBM without mucin allowing recovery of <10 CFU/mL and DBM + 0.2% mucin allowing recovery of 6.1 X 10^6^ CFU/mL.*

*
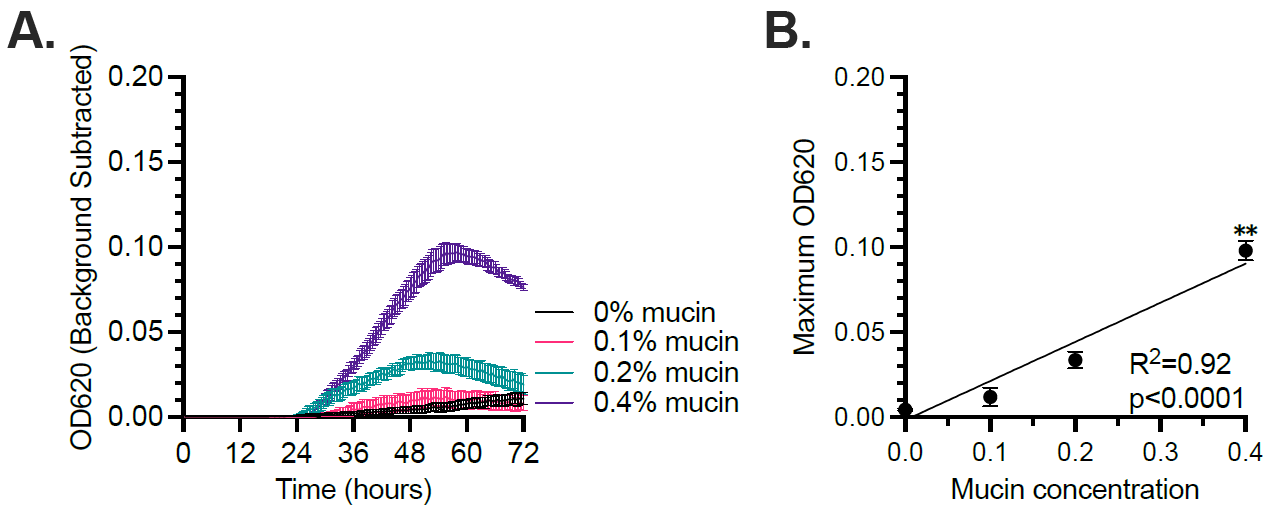
*

***Figure S3****: Repeat growth of L. pullorum ET540 in DBM + mucin. L. pullorum ET540 was grown in DBM + 0.2% mucin for 24 hr, diluted 5% v/v in fresh DBM containing the indicated mucin concentrations, and (****A****) growth over time in culture and (B) maximum OD_620_ relative to mucin concentration were plotted. In (A), mean ± SEM were plotted for triplicate cultures at each time point. In (****B****), mean ± SEM were plotted for triplicate cultures at each mucin concentration. Linear regression was used to determine the goodness of fit (R2) and significance of slope deviation from zero, with p<0.05 reported. Significance of differences in maximum OD_620_ values at each mucin concentration relative to no mucin was determined by one-way ANOVA with Brown-Forsythe correction for unequal variances and Dunnett’s T3 correction for multiple comparisons. **, p<0.01.*

*
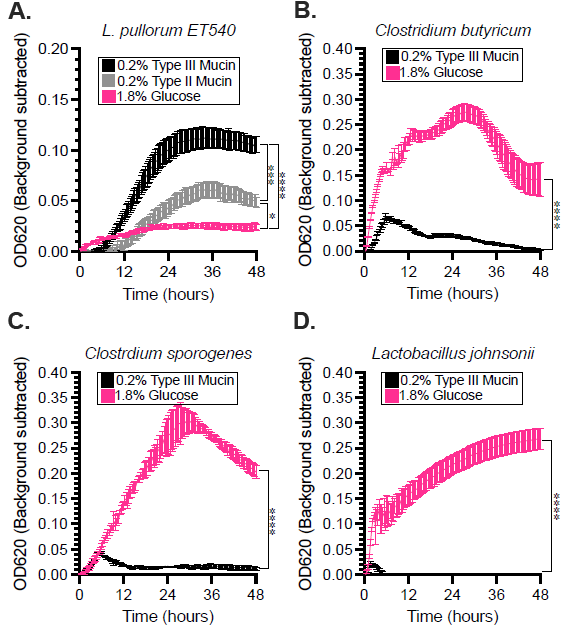
*

***Figure S4****: Growth of ET540 along with non-mucin metabolizing species in mucin and glucose. Strains were grown in their preferred growth medium for 16 (Clostridium butyricum ATCC 19398 [BHIS], C. sporogenes ATCC 3584 [BHIS], Lactobacillus johnsonii NC533 [MRS]) or 24 (L. pullorum ET540 [DBM + 0.2% Type II PGM] hours, then diluted 5% v/v in fresh DBM containing 0.2% Type III PGM, 1.8% glucose, or 0.2% Type II PGM (ET540 only). Growth over 72 hr in culture was plotted for (****A****) L. pullorum ET540, (****B****) C. butyricum, (****C),*** *C. sporogenes, or (****D****) L. johnsonii. Data represent mean ± SD for triplicate cultures at each time point. Statistical significance of difference in the areas under the curve was determined by one-way ANOVA with Tukey’s correction for multiple comparison (****A****) or two-tailed student’s t-test (****B****-****D****); *, p<0.05; ***, p<0.001; ****, p<0.0001.*

*
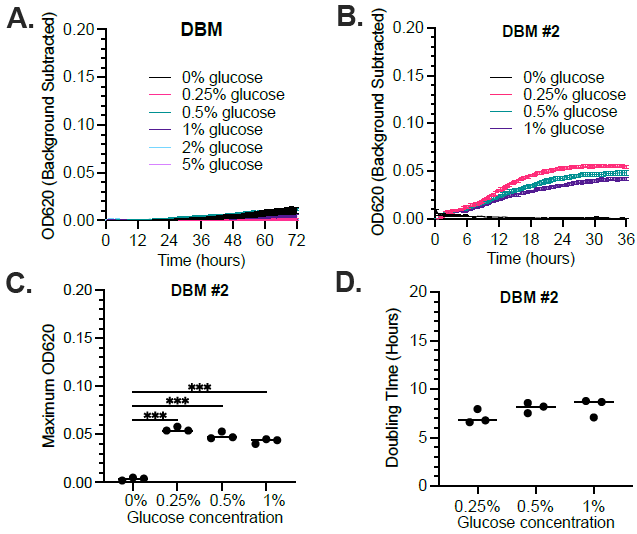
*

***Figure S5****: Growth of L. pullorum ET540 in defined medium with glucose as the sole carbon source. (****A****) L. pullorum ET540 was grown in DBM + 0.2% mucin for 24 hr, diluted 5% v/v in fresh DBM containing the indicated glucose concentrations, and mean ± SEM was plotted for triplicate cultures at each time point. In (****B****)-(****D****), L. pullorum ET540 was grown in DBM + 0.2% mucin for 48 hr, diluted 5% v/v in fresh DBM #2 containing the indicated glucose concentrations, and (****B****) growth over time in culture, (****C****) maximum OD_620_ relative to glucose concentration, and (****C****) doubling time from 2-24 hr for each glucose concentration were plotted. In (****A****) and (****B****), mean ± SEM were plotted for triplicate cultures at each time point. In (****C****) and (****D****), each point represents a replicate with the line indicating the median. Significance of difference in maximum OD_620_ from no added glucose and doubling time between mucin concentrations was determined by one-way ANOVA with Brown-Forsythe correction for unequal variances and Dunnett’s T3 correction for multiple comparisons. ***, p<0.001.*


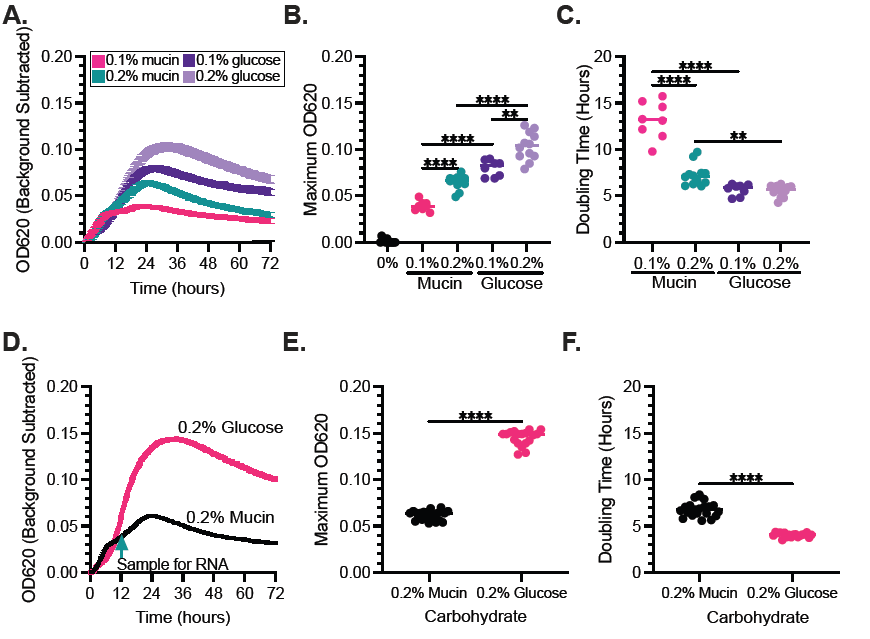


***Figure S6:*** *Growth of L. pullorum strains in modified defined basal medium yields growth with mucin or glucose. In (****A****)-(****C****), L. pullorum ET540 was grown in DBM + 0.2% mucin for 48 hr, diluted into fresh DBM #2 medium with 0.1% or 0.2% mucin or glucose, and (****A****) growth over time in culture, (****B****) maximum OD_620_ relative to mucin concentration, and (****C****) doubling time from 2-24 hr for each mucin or glucose concentration were plotted. In (****A****), mean ± SEM values were plotted for replicate cultures at each time point. In (****B****), significance of differences in maximum OD_620_ between 0% and 0.1% or 0.2% of mucin or glucose, between 0.1% and 0.2% of the same substrate, or between the same percentage of different substrates (e.g., 0.1% mucin and 0.1% glucose), was determined by one-way ANOVA with Brown-Forsythe correction for unequal variances and Dunnett’s T3 correction for multiple comparisons. All cultures grown in medium with mucin or glucose were significantly higher than with no added carbohydrate (p<0.0001, not shown). In (****C****), significance of differences in doubling times between 0.1% and 0.2% of the same substrate or between the same percentage of different substrates was determined by one-way ANOVA with Brown-Forsythe correction for unequal variances and Dunnett’s T3 correction for multiple comparisons. In (****D****)-(****F****), L. pullorum was grown under the same conditions as in (****A****)-(****C****) in 0.2% mucin or 0.2% glucose to collect samples for transcriptomics. In (****D****), growth over time in culture was plotted as the mean ± SEM and the time of sample collection for RNA extraction is indicated by an arrow. In (****E****) and (****F****), each point represents a replicate with the line indicating the median. Significance of differences in maximum OD_620_ or doubling time between cells grown in mucin or glucose was determined by two-tailed student’s t-test with Welch’s correction for unequal variances.*

*
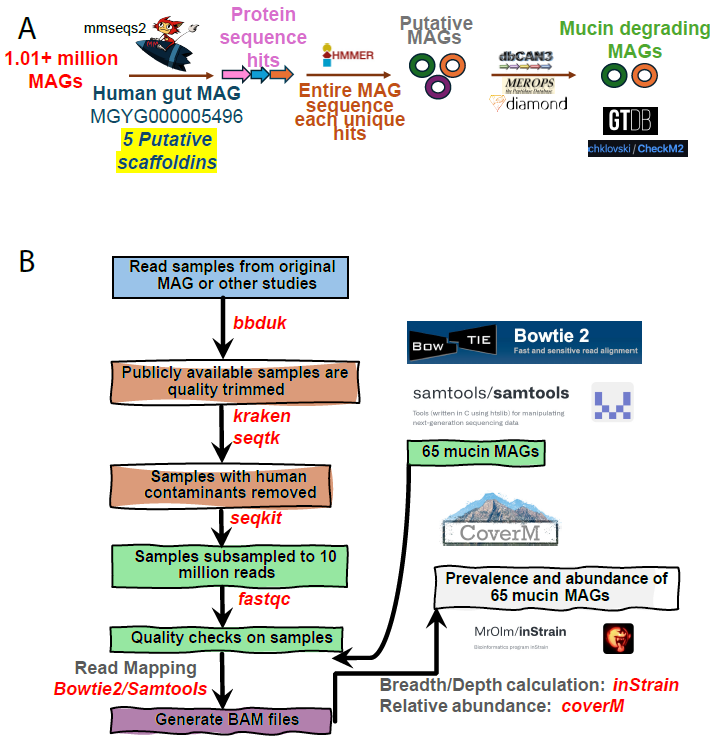
*

***Figure S7:*** *Computational workflows and tools.* *(****A****) Identification of the 65 MAGs. (****B****) and Read mapping to determine MAG abundances in their original samples and MAG prevalence in 2,897 fecal samples of different human and animal hosts.*

*
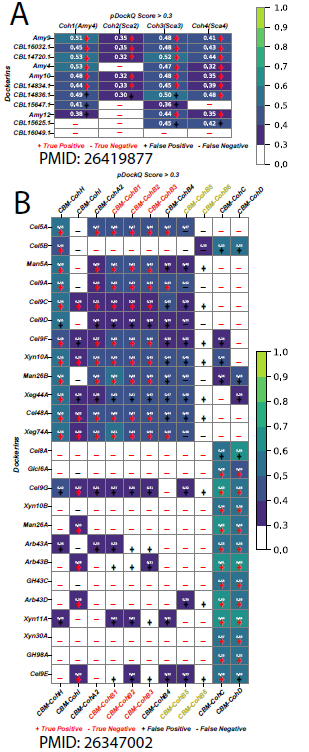
*

***Figure S8:*** *AlphaFold3 predicted functional Coh-Doc interactions.* *Dockerin and cohesin modular sequence pairs were used as input to AlphaFold3 for 3D structure and interaction predictions. The resulting PDB files served as input to FoldDock to calculate the* *pDockQ scores. Scores larger than 0.3 are shown.* *(****A****) Protein-protein interactions (PPIs) are predicted between cohesins (rows) and dockerins (columns) in GHs of Ruminococus bromii L2-63. “+” and “-“ indicate the presence and absence of experimentally characterized PPIs (85).* *(****B****) PPIs are predicted between cohesins (rows) and dockerins (columns) in GHs of Ruminococcus champanellensis. “+” and “-“ indicate the presence and absence of experimentally characterized PPIs (86).*

*
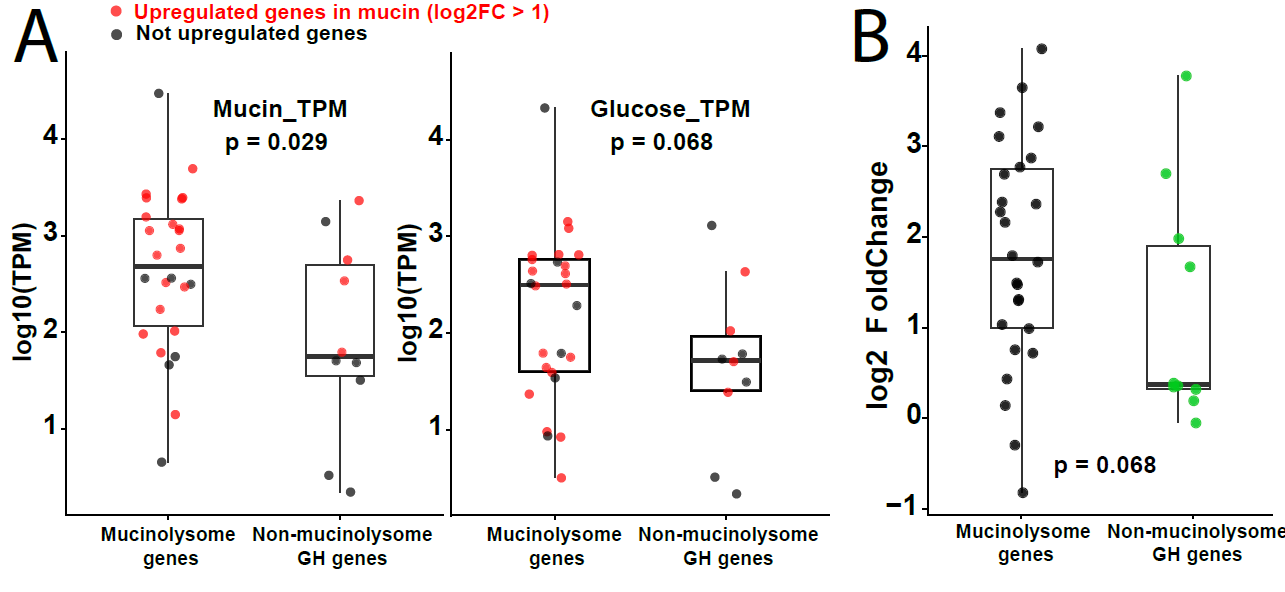
*

***Figure S9:*** *Comparison of expressions of mucinolysome genes (GHs + Scaffoldins) and non-mucinolysome GH genes.* *(****A****) Expression values in mucin samples (left) and gluclose samples (right). (****B****) Up-regulation fold changes in mucin samples relative to gluclose samples. P-values were calculated with one-sided Wilcoxon rank-sum tests.*

**Supplementary tables**

**Table S1: 65 genomes that encode mucinolysomes**

**Table S2: Functional domains in proteins of mucinolysomes of 65 genomes**

**Table S3. CAZymes in five genomes**

**Table S4: PPIs predicted for Coh-Doc pairs in *C. perfringens* ATCC 13124**

**Table S5: PPIs predicted for Coh-Doc pairs in Lpuc ET540**

**Table S6: Chemically defined media composition**

**Table S7: Gene expression of all genes in ET540**

**Table S8: All MAGs searched in this study**

**Table S9: SRA samples of 65 genomes**

**Table S10: 2,897 fecal samples of different animal hosts**
